# Absolute Quantification of Brain Deuterium Metabolic Imaging in Healthy Volunteers and Glioblastoma Patients at 7T

**DOI:** 10.1002/mrm.70308

**Published:** 2026-02-23

**Authors:** Jabrane Karkouri, Masha Novoselova, Sarah Miller, Minghao Zhang, Alexandra Constantinou, Carina Graf, Özlem Ipek, Daniel Atkinson, Brandon Tramm, Scott Schillak, Richard Mair, Kevin Brindle, Christopher T. Rodgers

**Affiliations:** ^1^ Wolfson Brain Imaging Centre University of Cambridge Cambridge UK; ^2^ School of Biomedical Engineering and Imaging Science, King's College London London UK; ^3^ Virtumed, LLC Minneapolis Minnesota USA; ^4^ Cancer Research UK Cambridge Institute University of Cambridge, Li Ka Shing Centre Cambridge UK; ^5^ Department of Biochemistry University of Cambridge Cambridge UK

**Keywords:** array head coils, brain metabolism, brain tumor, deuterium metabolic imaging, glioblastoma

## Abstract

**Purpose:**

We present a method for absolute quantification of deuterated metabolites in vivo at 7T. We describe acquisition protocols and an analysis pipeline compatible with 7T Terra MRIs. We apply them using a ^2^H/^1^H receive array in healthy volunteers and glioblastoma patients.

**Methods:**

B_1_^+^/B_1_^−^ maps from multiple CSI scans in a uniform phantom were used to derive calibrated coil weights for absolute quantification. We validated this in phantoms with known deuterated compounds. ^2^H MRSI was performed in twelve healthy volunteers (two post‐[6,6‐^2^H_2_]‐glucose) and five treatment‐naive glioblastoma patients (all post‐[6,6‐^2^H_2_]‐glucose). Spectra were fitted with OXSA. We compared Glx/Lac between tumor and normal‐appearing brain using linear mixed‐effects models.

**Results:**

Measured B_1_^+^ maps were 0.82 ± 0.19 μT/√W across the whole phantom. Natural abundance deuterium in water was 8.96 ± 0.7 mmol/L. Absolute maps of HDO, Glc, Glx, and Lac were acquired following [6,6‐^2^H_2_]glucose. Rate maps showed higher Lac production in tumors (2.3 μmol/L/min, SE = 0.87) compared with normal‐appearing regions (1.0 μmol/L/min, SE = 0.36; *p* < 0.01) and healthy brain (0.5 μmol/L/min, SE = 0.17; *p* < 0.01). Glx production was lower in tumors (3.8 μmol/L/min, SE = 0.44) relative to normal‐appearing contralateral regions (6.0 μmol/L/min, SE = 0.36; *p* < 0.001) and healthy brain (9.2 μmol/L/min, SE = 0.61; *p* < 0.001).

**Conclusion:**

We demonstrate robust absolute quantification for human 7T DMI. Glioblastoma tumors showed elevated Lac and reduced Glx labeling relative to normal brain, with inter‐patient heterogeneity consistent with an existence of different metabolic subtypes.

## 1 Introduction

Altered brain energy metabolism is a hallmark of prevalent diseases, including neurodegenerative disorders and cancer [1, 2, 3, 4, 5]. Insufficient cellular energy impairs neuronal function, leading to cell death during aging and as a result of neurodegenerative disease. Therapies aimed at restoring brain energy metabolism—such as oral supplementation with NAD^+^ or exogenous ketones, or intermittent fasting—are actively being investigated to treat Parkinson's disease and other disorders [6, 7, 8, 9]. Tumors, by contrast, often display fundamentally different metabolism, with a preference for glycolysis [5, 10, 11, 12, 13]. Non‐invasive characterization of such metabolic changes could enable personalized treatment selection [14]. Alternatively, tumor metabolism itself is an attractive therapeutic target; recent reviews highlight glycolysis, glutaminolysis, and mitochondrial pathways as key precision oncology targets [11, 15].

Glioblastoma, the most aggressive brain cancer, is metabolically heterogeneous. Spatial transcriptomics and mass spectrometry imaging of ^13^C‐glucose–infused patients reveal glycolytic and oxidative subtypes [16, 17, 18, 19]. These can coexist at the centimeter scale, suggesting they may be resolvable by clinical metabolic imaging. Because the glycolytic subtype is radiation‐resistant and carries a poorer prognosis, imaging could inform prognosis and treatment selection. Clinical trials of energy‐modifying therapies likewise require robust, non‐invasive metabolic endpoints to permit trials in economically‐viable cohorts.

Magnetic resonance spectroscopy (MRS) and spectroscopic imaging (MRSI) provide powerful in vivo tools to probe brain metabolism [20, 21, 22]. A particularly promising emerging MRSI approach, called deuterium metabolic imaging (DMI), uses deuterium‐labeled substrates to track metabolic fluxes in real time. DMI was introduced in 2017 at 16.4T [23] and has subsequently been applied in vivo by several groups [23, 24, 25, 26, 27, 28, 29, 30, 31, 32, 33, 34, 35, 36, 37, 38, 39, 40, 41, 42, 43].

DMI reveals regional differences in the balance between glycolytic and oxidative glucose metabolism. ^2^H‐MRSI follows metabolism of [6,6‐^2^H_2_]‐glucose into glutamate/glutamine (Glx) via the TCA cycle and into lactate (Lac) via glycolysis, as pioneered by de Feyter and de Graaf [13, 23, 42, 44, 45]. This provides unique insights into metabolic reprogramming and may serve as a radiation‐free alternative to ^18^FDG‐PET, as shown recently in Alzheimer's disease [26].

Preclinical work supports DMI's potential for monitoring treatment response [10, 14, 46, 47, 48, 49], for grading tumors [50, 51, 52] and to determine subtypes of glioblastoma xenografts by quantifying [6,6‐^2^H_2_]‐glucose partitioning between Glx and Lac [14]. Ultra‐high‐field MRI improves both SNR and also spectral separation (between HDO, Glc, Glx, and Lac), as demonstrated by studies at 9.4T [53] and 7T [27, 28, 35, 36, 43, 54, 55, 56] compared to 3T [31, 33, 34, 41, 57].

Here, we focus on quantifying deuterium metabolites at 7T in healthy volunteers and glioblastoma patients. We adapted a method for *absolute quantification* (finding metabolite concentrations in mol/L) that was originally developed by Purvis et al. [58] for liver 7T ^31^P‐MRS. We validated it in phantoms and in healthy volunteers without administration of a deuterated tracer. Then, we applied the method to quantify the temporal dynamics of HDO, Glc, Glx, and Lac concentrations after oral [6,6‐^2^H_2_]glucose. We compared healthy tissue in volunteers with tumor‐containing and normal‐appearing regions in patients to reveal inter‐patient and intra‐tumor metabolic heterogeneity.

## 2 Theory

### 2.1 Quantitation Framework

The key requirements for quantitative MRSI with a receive array are: (i) combining signals from array elements appropriately for quantitation, (ii) fitting spectral peaks, and (iii) correcting for metabolite and reference phantom relaxation.

We adapt “Method 1: Phantom Replacement” from Purvis et al. which was originally developed for 7T ^31^P‐MRS of the human liver [58]. This relies on acquiring data from a conductivity‐matched phantom of known concentration and T_1_ with a matched protocol for calibration.

### 2.2 B_1_ ^+^ Mapping

The Phantom Replacement method requires knowledge of the coil's B_1_^+^ and B_1_^−^ fields. Following Purvis et al. [58], we measured these using Variable Flip Angle (VFA) B_1_^+^ mapping on a uniform phantom [59]. Multiple CSI scans were acquired with $N_{V}=9$ RF excitation voltages (V1,V2,…). For each voxel, all the single‐element spectra were concatenated to form a joint spectrum:

(1) $$S\left(r\right)=\left(\begin{array}{lll} S_{V_{1},ch_{1}} & \ldots & S_{N_{V},ch_{1}}\\ \vdots & & \vdots \\ S_{V_{1},N_{c}} & \ldots & S_{N_{V},N_{c}} \end{array}\;\right)$$

of size $N_{c}\times \left(N_{V}N_{s}\right)$, where $N_{c}$ is the number of receive elements and $N_{S}$ is the number of points in each spectrum. The noise covariance Ξ was estimated from a no‐RF prescan. Whitened Singular Value Decomposition (WSVD) was then applied in each voxel to compute $N_{c}\times 1$ coil weight vectors w(r) jointly for all the CSI scans, and maximum likelihood combined spectra $S_{j}\left(r\right)$.

The combined spectrum with maximum signal was fitted in AMARES [60] to obtain a model spectrum $S_{M}\left(r\right)$. Using linear least squares (“∖” in MATLAB), complex single‐element amplitudes $A_{k,j}\left(r\right)=S_{k,j}\left(r\right)\setminus S_{M}\left(r\right)$ and combined amplitudes $A_{j}\left(r\right)=S_{j}\left(r\right)\setminus S_{M}\left(r\right)$ were derived for each element k and voltage j.

Using the known T_R_ (1000 ms) and phantom T_1,P_ (355 ms), voxel‐wise $B_{1}^{+}$ maps were obtained by nonlinear least‐squares fitting (lsqcurvefit, MATLAB) to: 

(2)

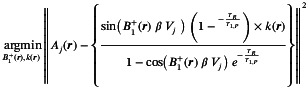


where $B_{1}^{+}\left(r\right)$ is the transmit field strength per volt in units of μT/V_RMS_, and β=γH2×∫0TPf(t)dtaccounts for the deuterium gyromagnetic ratio γH2 and the RF pulse shape f(t) integrated over the pulse duration $T_{P}$, $T_{R}$ is the repetition time, $T_{1,P}$ the phantom P ^2^H T_1_ and k(r) is a scaling factor representing both coil receive sensitivity and the phantom concentration [61].

### 2.3 B_1_ ^−^ Mapping

According to the Roemer signal model [62], the signal in the *k*th element depends on the sample magnetization and that element's receive sensitivity $B_{1}^{-}$. The magnetization depends on the ^2^H concentration (known in the phantom), $B_{1}^{+}$ and the pulse sequence. We include in “receive sensitivity” all fixed factors (cable losses, preamplifier/channel gains), which remain identical for reference and main scans. The overall phase of $B_{1}^{-}$ was defined by assuming the spectrum at the voltage giving maximum signal was real and positive.

(3) $$\underset{B_{1}^{+}\left(r\right),k\left(r\right)\;}{\text{argmin}}{\left\|A_{j,k}\left(r\right)-\left\{\frac{\sin\left(B_{1}^{+}\left(r\right)\;\beta \;V_{j}\;\right)\;\left(1-e^{-\frac{T_{R}}{T_{1,P}}}\right)}{1-\cos\left(B_{1}^{+}\left(r\right)\;\beta \;V_{j}\right)\;e^{-\frac{T_{R}}{T_{1,P}}}}\right\}B_{1k}^{-}\left(r\right)\right\|}^{2}$$

This was solved by linear least squares (“∖” in MATLAB). Effectively, the scaling k(r) from the $B_{1}^{+}$‐mapping is distributed across receive elements, with relative phases captured since $B_{1}^{-}$ is complex.

### 2.4 Uniform Sensitivity Coil Combination

The joint WSVD weights $w_{k}$ at position r are mathematically equivalent to Roemer's uniform sensitivity weights (Equation 27, [62]):

(4) $$w_{k}\left(r\right)=\frac{\Xi^{-1}B_{1}^{-}\left(r\right)}{B_{1}^{-}{\left(r\right)}^{ϯ}\;\Xi^{-1}B_{1}^{-}\left(r\right)\;}$$

Roemer weights capture the complex scaling from spatially varying coil sensitivity, whereas WSVD—being data‐driven—leaves an arbitrary unknown overall sensitivity.

For quantitative analysis, we apply the reference‐phantom‐derived weights to combine per‐element spectra from the main scan (e.g., an in vivo scan to be calibrated). This assumes that $B_{1}^{-}$ and noise covariance Ξ are similar between the reference and main scans, which holds if conductivity and coil loading are comparable. The combined spectra are then fitted with AMARES, giving metabolite amplitudes ${\hat{S}}_{m}\left(r\right)=\sum_{k=1}^{N}w_{k}\left(r\right)S_{m,k}\left(r\right)$ for the main scan and ${\hat{S}}_{P}\left(r\right)=\sum_{k=1}^{N}w_{k}\left(r\right)S_{P,k}\left(r\right)$ for the reference phantom.

Following Purvis et al. (Equation 10), the metabolite concentration is:

(5) $$\left[m\right]\left(r\right)=\frac{F_{m}\left(r\right)\;\sum_{k=1}^{N_{c}}w_{k}\left(r\right)S_{m,k}\left(r\right)}{F_{P}\left(r\right)\;\sum_{k=1}^{N_{c}}w_{k}\left(r\right)S_{P,k}\left(r\right)}\left[P\right]\left(r\right)=\frac{F_{m}\left(r\right)\;{\hat{S}}_{m}\left(r\right)}{F_{P}\left(r\right)\;{\hat{S}}_{P}\left(r\right)}\left[P\right]\left(r\right)$$

where $F_{m}$ and $F_{p}$are saturation‐correction factors for metabolite m and the reference phantom, which has concentration [P], and $N_{c}$ is the number of receive elements. For a spoiled CSI sequence:

(6) $$F_{m}\left(r\right)=\frac{1-\cos\left(B_{1}^{+}\left(r\right)\;\beta \;V_{j}\;\right)e^{-\frac{T_{R}}{T_{1,m}}}}{\sin\left(B_{1}^{+}\left(r\right)\;\beta \;V_{j}\;\right)\left(1-e^{-\frac{T_{R}}{T_{1,m}}}\right)}$$

where $T_{1,m}$ is the $T_{1}$of metabolite m (or phantom compound).

## 3 Methods

### 3.1 Hardware

**FIGURE 1:**
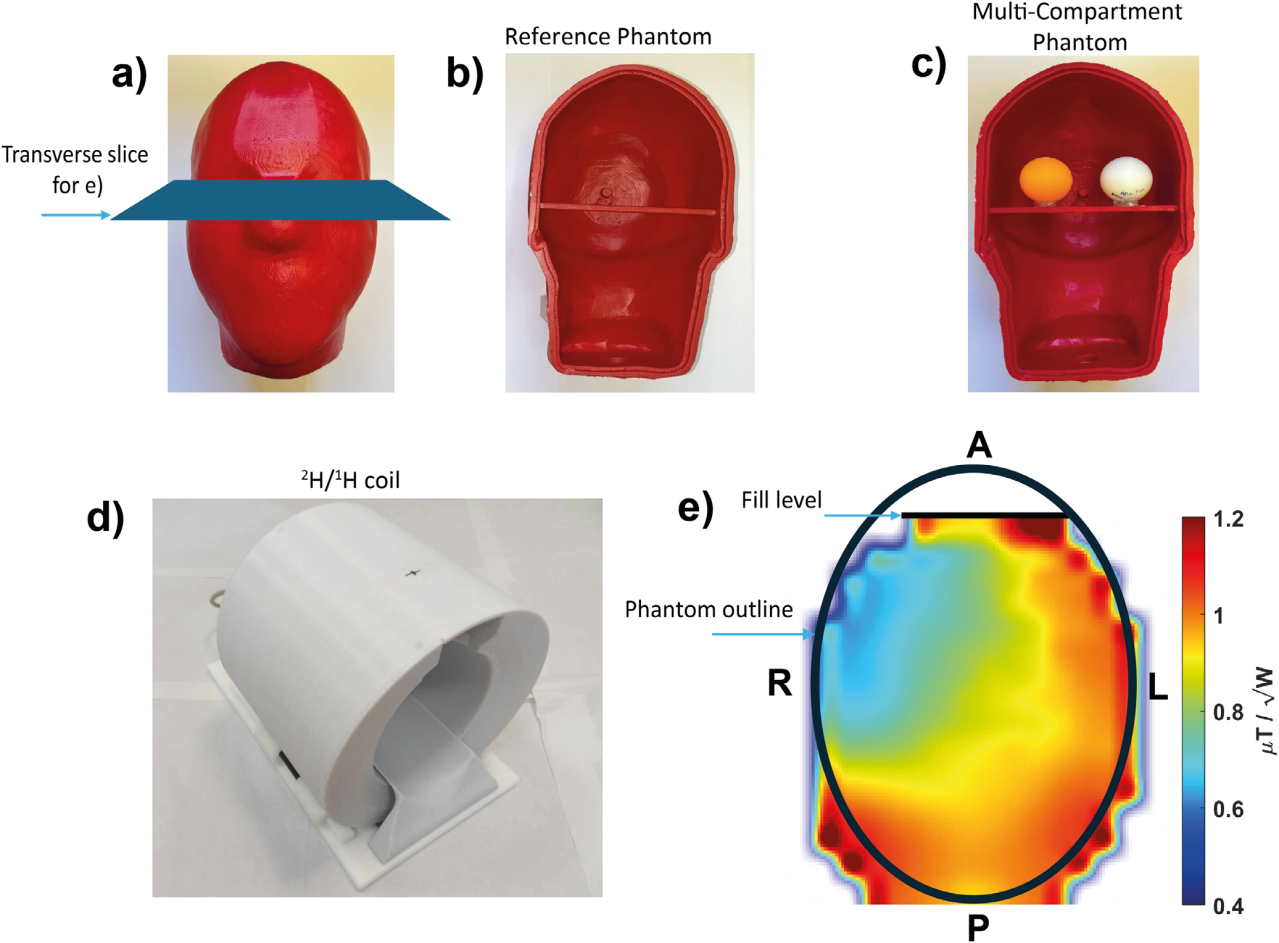
Custom 3D‐printed head‐shaped phantoms for calibration and validation. (a) Photograph of the phantom housing. (b) Reference phantom filled with deuterium oxide and water. (c) Multi‐compartment phantom containing two table tennis balls containing aqueous solutions of Lac and Fum, with the remaining space filled with deuterium oxide and water. (d) Photograph of the dual‐tuned 7T ^2^H/^1^H head coil used for this study. (e) B_1_^+^ map with the reference phantom (b) plotted for the mid‐transverse slice illustrated in (a).

All experiments were performed using a Magnetom Terra 7T MRI (Siemens, Erlangen, Germany) with a dual‐tuned ^2^H/^1^H head coil (Virtumed LLC, Minneapolis, MN). Figure 1d shows a photograph of the coil, which comprises a 15‐element Transverse Electromagnetic mode (TEM) resonator for ^2^H transmit (one rung is left out to allow visual stimulus; rungs approx. 17.7 cm long, 4.7 mm radius), 16× ^2^H receive loops (17.4 cm × 6.8 cm), and 4× ^1^H transmit/receive loops driven in quadrature (2× ^1^H loops 17.4 cm × 14.4 cm and 2× ^1^H loops 17.4 cm × 4.8 cm).

### 3.2 Reference Phantom

We 3D‐printed a custom head‐shaped phantom (Figure 1a) based on the design by Shajan et al. [63], to which we added a threaded bottle neck and cap, and a support for the inner compartments (table tennis balls). It was printed from polylactic acid (80% infill, Bambu 3D printer, Shenzhen, China) and waterproofed with four coats of red rubber spray (PlastiDip UK Limited, Petersfield, UK). The phantom was filled with 4.5 L ultrapure water (Electrolube, Ashby de la Zouch, UK), 51 g deuterium oxide (yielding an HDO concentration of 1.14 mol/L), 0.25 g Dotarem (Guerbet, Princeton, USA), 20.93 g sodium chloride, and 8.4 g sodium benzoate. Chemicals were obtained from Sigma‐Aldrich and used as supplied. Conductivity and permittivity were measured with a DAK12‐TL probe beam (Schmid & Partner Engineering AG, Zurich, Switzerland) and were 0.98 S/m and 82.81, comparable to tissue values [64, 65], where gray matter is 0.47 S/m, white matter 0.27 S/m and cerebrospinal fluid 2.04 S/m.

### 3.3 Quantitation Protocol

The scans needed to implement this method are:

1. Main MRSI scan (in vivo or phantom) to be quantified.
2. Reference phantom B_1_ ^+^ maps to fix $w_{k}\left(r\right)$ in Equation (4) and $B_{1}^{+}\left(r\right)$ in Equation (3)
3. Phantom T_1_ determination.
4. Reference scan with matched protocol to determine $F_{P}\left(r\right)$and ${\hat{S}}_{P}\left(r\right)$in Equation (5)

Data from steps 2–3 can be reused to quantify many MRSI scans providing their protocols match, including having identical position and orientation for their MRSI matrices.

### 3.4 B_1_ ^+^ and B_1_ ^−^ Maps

Reference phantom B_1_ ^+^ and B_1_ ^+^ maps were obtained by acquiring nine 3D CSI scans with 4 ms rectangular excitation pulses having peak voltages ranging from 25 to 225 V in 25 V steps, 1 s TR, 16 × 16 × 8 matrix, 5 kHz spectral bandwidth, 1024 points, and acquisition weighting with 1 average at *k* = 0. The total acquisition time was 1 h 30 min.

### 3.5 Validation of Absolute Quantification

The reference phantom (Figure 1a,b) was scanned as part of the “Reference scan” with $T_{R}$ = 250 ms, 16 × 16 × 8 matrix, FOV = 220 × 200 × 320 mm^3^, 1 ms block excitation pulse at 210 V, 5 kHz spectral bandwidth over 1024 points, 6 averages, and acquisition weighting, giving a total time of 5 min. A second scan (i.e., the “main scan” for the purpose of this test) was performed on another day with identical parameters and grid position.

A second multi‐compartment phantom was built using the same head‐shaped shell but with two table‐tennis balls glued inside (Figure 1c). Each contained 50 mL ultrapure water and 0.23 g sodium chloride. One was supplemented with [2,3‐^2^H_2_]fumaric acid (CLM‐1529; 0.99 g; MW 118.1 g/mol; 0.25 mol/L; deuterium concentration 0.5 mol/L) and the other with sodium L‐[3,3,3‐^2^H_2_]lactate (DLM‐9071; 1 g; MW 115.03 g/mol; 0.26 mol/L; deuterium concentration 0.78 mol/L). Both were obtained from CK Isotopes Limited (Leicester, United Kingdom). The bulk head volume contained 9 g D_2_O and water, giving an HDO concentration of 0.2 mol/L.

To minimize voxel partial‐volume effects, we used a 20 × 20 × 16 matrix while keeping other parameters identical, for a total acquisition time of 29 min 12 s. A matching calibration scan of the reference phantom was also acquired at this resolution. Following Purvis et al. [58], B_1_ maps were interpolated to this grid using MATLAB's *imresize3.m* (bilinear interpolation).

#### 3.5.1 T_1_ Measurements

Separate measurements were performed to determine the *T*_1_ values for each metabolite in each phantom, that is, HDO in the reference phantom, and HDO, Lac, and Fum in the multi‐compartment phantom.

The reference phantom was scanned alone, each table‐tennis ball was scanned individually, and finally the complete multi‐compartment phantom was scanned after assembly. The $T_{1}$ values of each metabolite were measured using non‐localized inversion recovery scans and fitted to a 3‐parameter model. The inversion‐recovery acquisitions had inversion times that were incremented from 25 ms to 8000 ms, 10 s TR, 1 ms rectangular excitation pulses and 2 ms rectangular inversion pulses both set to 150 V_rms_.

### 3.6 In Vivo Data Acquisition

#### 3.6.1 Participants

Twelve healthy volunteers (6 females, 6 males, 29–58 years) and five glioblastoma patients (2 females, 3 males, 53–67 years) gave written consent. Ethics approvals were obtained from the University of Cambridge HBREC (HBREC.2021.08) and the London Camden & King's Cross REC (21/PR/0828).

#### 3.6.2 Study Protocol

Ten healthy volunteers were scanned using natural‐abundance HDO only. The remaining two volunteers and all patients fasted ≥ 4 h before receiving a glucose drink (0.75 g/kg body weight, max 60 g) of 6,6′‐[^2^H_2_]glucose in ≈200 mL water prior to the 7T scan. Patients also underwent a 3T clinical scan during the same visit. All patients were treatment naïve, that is, they had not yet received any treatment or surgery. For the two healthy volunteers, the imaging protocol included a baseline (pre‐ingestion) ^2^H‐DMI scan immediately before oral administration of the ^2^H‐glucose drink. The five GBM patients did not undergo a baseline acquisition due to clinical time constraints. Their DMI scans began after glucose ingestion.

#### 3.6.3 7T MRI Protocol

Participants were scanned lying head‐first supine, with lights switched off at the main circuit breaker to reduce noise at the ^2^H frequency (Figure SI1). MRI acquisition began with ^1^H gradient‐echo scouts (TE = 3.6 ms, TR = 8.6 ms, FOV = 400 mm^2^, 1.56 × 1.56 mm^2^ in‐plane, 5 mm slice, 17° flip angle) followed by T_1_‐weighted images (TE = 1.8 ms, TR = 1 s, FOV = 280 × 245 × 192 mm^3^, 1.09 × 1.09 mm^2^ in‐plane, 1 mm slice, 5° flip angle).

B_0_ shimming was performed with the vendor's “GRE‐Brain” tool, and post‐shim maps were acquired with dual‐echo GRE (TE = 1.02/3.06 ms, TR = 18 ms, FOV = 256 × 256 × 256 mm^3^, 4 mm in‐plane, 4 mm slice, 7° flip angle).

^2^H MRSI data were collected in blocks by repeating a UTE‐CSI [66] sequence with 6.9 mL nominal resolution (16 × 16 × 8 matrix, FOV = 220 × 200 × 320 mm^3^, TR = 250 ms, 1 ms rectangular pulse at 210 V_RMS_, 5 kHz bandwidth), giving 4 min 59 s per measurement. The CSI grid and parameters matched those of the phantom scans and were fixed across all participants.

#### 3.6.4 3T Acquisition Protocol

The five glioblastoma patients also underwent 3T imaging (Prisma, Siemens, Erlangen, Germany) using a standard brain protocol [67, 68] including MPRAGE, T_2_ SPACE, T_2_‐weighted turbo spin echo, and diffusion‐weighted EPI, all following Gadovist contrast administration (Bayer New Zealand Limited, New Zealand).

### 3.7 Post‐Processing

Anatomical 3T images were co‐registered to the 7T GRE scan using an affine transformation with 12‐degrees of freedom and mutual information (imregtform, MATLAB). Data were processed in MATLAB R2022b with an updated OXSA toolbox supporting Siemens 7T Terra scanners, incorporating AMARES [69].

**FIGURE 2:**
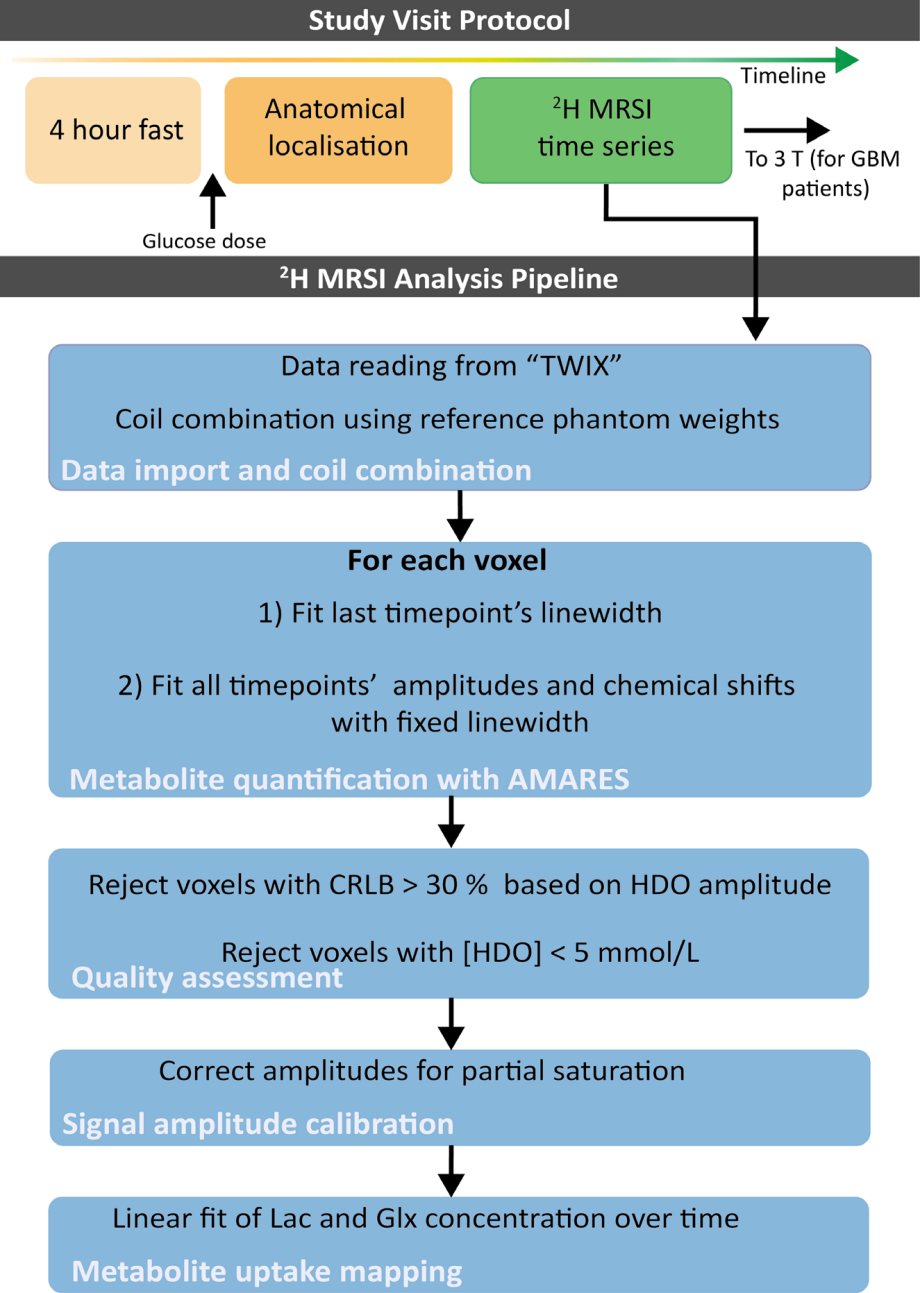
7T DMI protocol (top) and ^2^H MRSI processing steps (bottom). All participants fasted at least for 4 h prior to the scan, which included the uptake of a deuterated glucose drink, anatomical localization and a series of 3D CSI scans over time. The data were then exported for further analysis. A metabolite mask was generated from the HDO peak amplitude and its CRLB values before amplitudes were corrected for partial saturation. A pre‐ingestion (baseline) DMI scan was acquired in the two healthy volunteers but not in the GBM patients, who underwent post‐dose acquisitions only.

For ^2^H MRSI, analysis proceeded as follows (summarized in Figure 2):

1. Raw *k*‐space data (Siemens “TWIX” format) were Fourier transformed into image and frequency space.
2. For each voxel:
  - Multi‐channel signals were combined using Roemer weights calibrated on the reference phantom (see Section 2 above for details).
  - The last timepoint was quantified using prior knowledge of 4 Lorentzian peaks.
  - The prior knowledge was updated in memory to fix those linewidths before fitting data from all other timepoints.
  - Amplitudes were corrected for partial saturation using literature T_1_ values (Lac: 297 ms; Glc: 66 ms; Glx: 149 ms; HDO: 450 ms) [23, 24, 39].

#### 3.7.1 Quality Control and Thresholding

Cramér‐Rao Lower Bounds (CRLB) were computed to estimate uncertainty.

Mask #1 excluded voxels with [HDO] < 5 mmol/L, or HDO concentration CRLB > 30%.

Mask #2 was taken first from mask #1 and also excluded voxels with Lac/(Lac+Glx) concentration ratio CRLB > 30%. On average, 44% of the voxels from mask #1 were then discarded for mask #2 (ranging from 31% to 58%).

#### 3.7.2 Voxel‐Based ROI Analysis

To compare metabolism between healthy controls, tumor regions, and contralateral normal‐appearing brain, three ROIs were defined in patients. For each patient, a consultant neurosurgeon manually outlined the tumor region on 3T FLAIR images. ^2^H MRSI voxels overlapping the tumor region (ROI_tumor_), and equivalent contralateral voxels in normal‐appearing white matter (ROI_NAWM_) were selected based on the annotated 3T FLAIR images. In glucose‐ingesting healthy controls, a similar size ROI with voxels selected from the center of the brain was used (ROI_HV_). Voxel‐wise data within each ROI were averaged to yield an ROI mean value per region, per patient, per time point. These ROI‐mean data were used for all statistical analyses (see Figure SI2). We note that this approach does not constitute full anatomical ROI segmentation.

### 3.8 Time Series Analysis

Following the approach by Niess et al. [57], to test whether there were statistically significant changes in metabolite concentrations over time, we fitted Lac and Glx concentrations to a linear model [56, 57]:

(7) [m]∼1+time

This produces a summary of the rate of increase of Lac, that is, $\frac{d\left[\mathrm{Lac}\right]}{\mathrm{dt}},$and Glx, that is, $\frac{d\left[\mathrm{Glx}\right]}{\mathrm{dt}}.$ To capture the initial rate after glucose enters the bloodstream, we fitted datapoints 20–90 min after glucose uptake.

Maps of the relative metabolic flux toward lactate (i.e., lactate production rate divided by the sum of lactate and Glx production rates as a measure of the total glucose metabolism) $S_{\mathrm{Lac}}=\frac{d\left[\mathrm{Lac}\right]}{\mathrm{dt}}/\frac{d\left(\left[\mathrm{Lac}\right]+\left[\mathrm{Glx}\right]\right)}{\mathrm{dt}}$were then investigated with mask #2 (Section 3.7.1).

### 3.9 Statistical Analysis

#### 3.9.1 Regional Influence on Glx and Lac Temporal Dynamics

To assess whether tumor or normal‐appearing regions had different Glx and Lac kinetics, we used two complementary statistical approaches. First, we performed paired comparisons using the non‐parametric Wilcoxon Signed‐Rank Test [70], matching each tumor value with the corresponding value in normal‐appearing brain from the same patient at the same time point.

Second, we applied stepwise linear modeling (MATLAB “stepwiselm.m”) with the Akaike Information Criterion (AIC) to identify predictors of Glx or Lac concentrations. Candidate predictors included Patient ID, tissue type (tumor vs. normal‐appearing), and nuisance variables (age, gender, time). The best‐fitting models were then refined using linear mixed‐effects modeling (MATLAB “fitlme.m”) to account for repeated measurements within patients (Model 1) [71].

#### 3.9.2 Inter‐Patient Metabolic Heterogeneity in Tumor Regions

To examine inter‐patient metabolic variability, analysis was restricted to tumor regions and two linear mixed‐effects models were fitted. Model 2:

(8) [m]∼1+time+(1|PatientID)

This model assumes a fixed slope (d[m]/dt) across patients, with age, gender, and PatientID contributing variable intercepts.

And Model 3:

(9) [m]∼1+(time|PatientID)

where time is included as a random slope by patient, allowing each patient to have their own trajectory of metabolic change. Likelihood ratio tests [70] were used to assess whether including random slopes significantly improved model fit.

## 4 Results

### 4.1 Phantom Experiments

#### 4.1.1 B_1_ ^+^ Maps

Figure 1a,b shows the reference phantom used for the acquisition of the B_1_^±^ maps and for the validation of the absolute quantification methods. Figure 1e shows a mid‐transverse slice from the B_1_^+^ map. This shows a strong transmit field close to the edges of the phantom and a B_1_^+^ dropout in the right‐anterior region. The average B_1_^+^ for the mid‐transverse slice shown in Figure 1e is 0.90 ± 0.14 μT/√W(mean ± SD, with range: 0.61–1.25 $\frac{\mu T}{\sqrt{W}}$). Across the whole phantom it is 0.82 ± 0.19 μT/√W(range: 0.14–1.26 μT/√W).

#### 4.1.2 Validation of the Absolute Quantification Method

For the reference phantom, a $T_{1}$HDO of 697 ms was measured. For the multi‐compartment phantom, $T_{1}$Lac = 251 ms, $T_{1}$Fum = 314 ms and $T_{1}$HDO = 567 ms.

**FIGURE 3:**
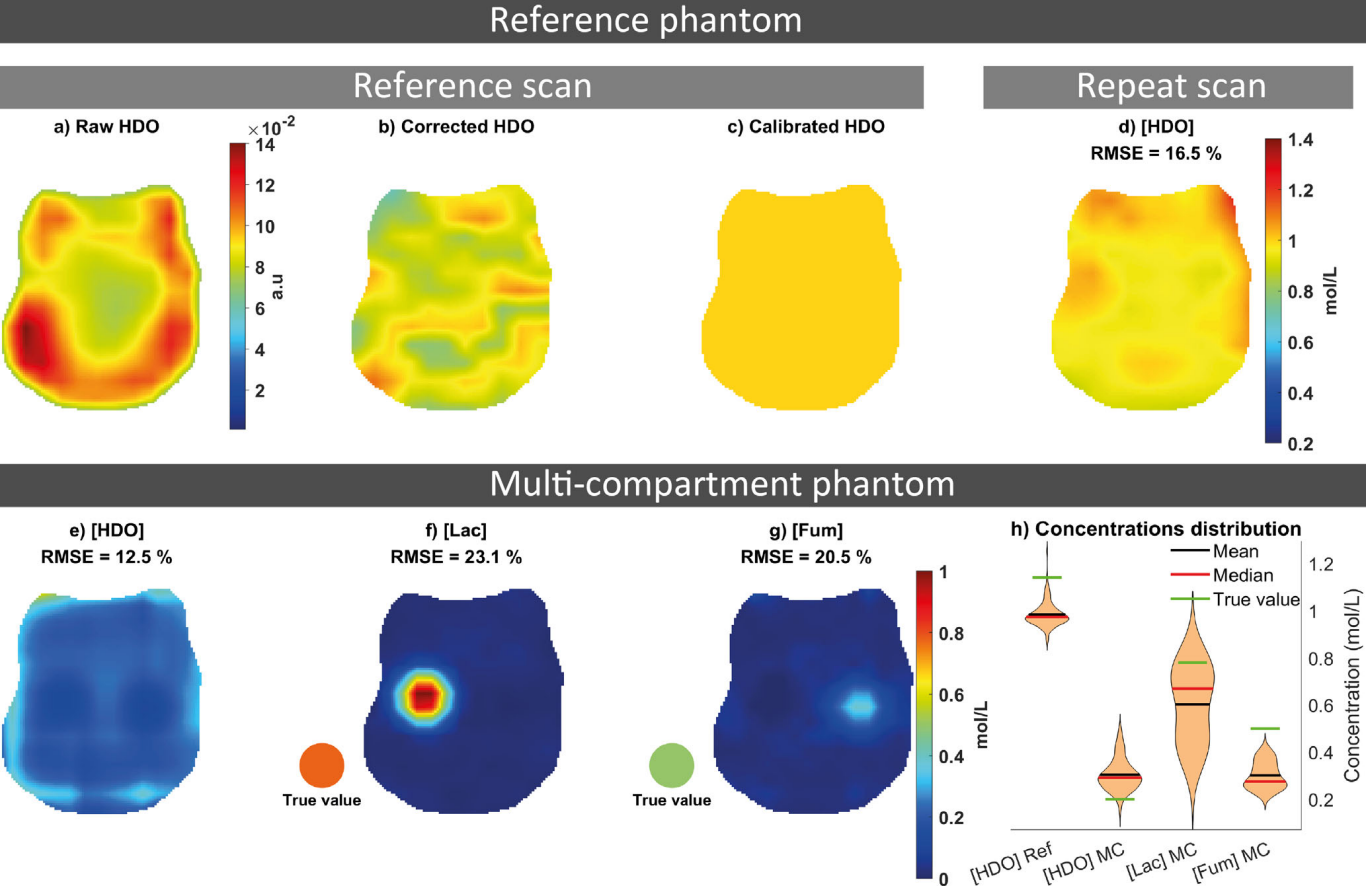
Phantom validation of absolute quantification concentration measurements. (a–d) reference phantom containing 1.14 mol/L HDO. (a) Raw HDO signal in the calibration scan without calibration for absolute quantification. (b) Corrected HDO signal for partial saturation in the calibration scan using Equation (5). (c) quantitative HDO map after calibration for absolute quantification in the calibration scan. (d) Quantitative HDO map after calibration for absolute quantification using a CSI scan acquired on a different date (repeat scan). Phantom validation of concentration measurements on the (e–h) multi‐compartment phantom containing 0.2 M HDO deuterons, 0.78 M Lac deuterons (in left ball) and, 0.5 M Fum deuterons (in right ball). [HDO], [Lac] and [Fum] metabolite maps are shown in (e), (f), and (g), respectively after calibration using Equation (5). (h) Distribution of the [HDO] values within the reference phantom, and of the [HDO], [Lac] and [Fum] within each whole 3D compartment of the multi‐compartment (MC) phantom.

Results validating the absolute quantification method are shown in Figure 3. For the calibration scan, the non‐calibrated HDO amplitude map is shown in Figure 3a, with saturation‐corrected and calibrated maps in Figure 3b,c. As expected, calibration yielded a uniform concentration matching exactly the phantom value. For the repeat scan, the calibrated [HDO] map (as per Equation 5, Figure 3d) gave an average concentration of 1.00 ± 0.05 mol/L (range 0.87–1.25 mol/L), a 12% error relative to the true value (1.14 mol/L).

In the multi‐compartment phantom (Figure 3e–g), average concentrations were: [HDO] 0.30 ± 0.07 mol/L (range 0.14–0.50 mol/L, 33% error), [Lac] 0.67 ± 0.18 mol/L (range 0.47–0.89 mol/L, 14% error), and [Fum] 0.30 ± 0.06 mol/L (range 0.25–0.39 mol/L, 40% error). Figure 3h shows the distribution of [HDO], [Lac], and [Fum] within each compartment of each phantom.

### 4.2 In Vivo DMI

#### 4.2.1 Natural Abundance Scans in Healthy Volunteers

**FIGURE 4:**
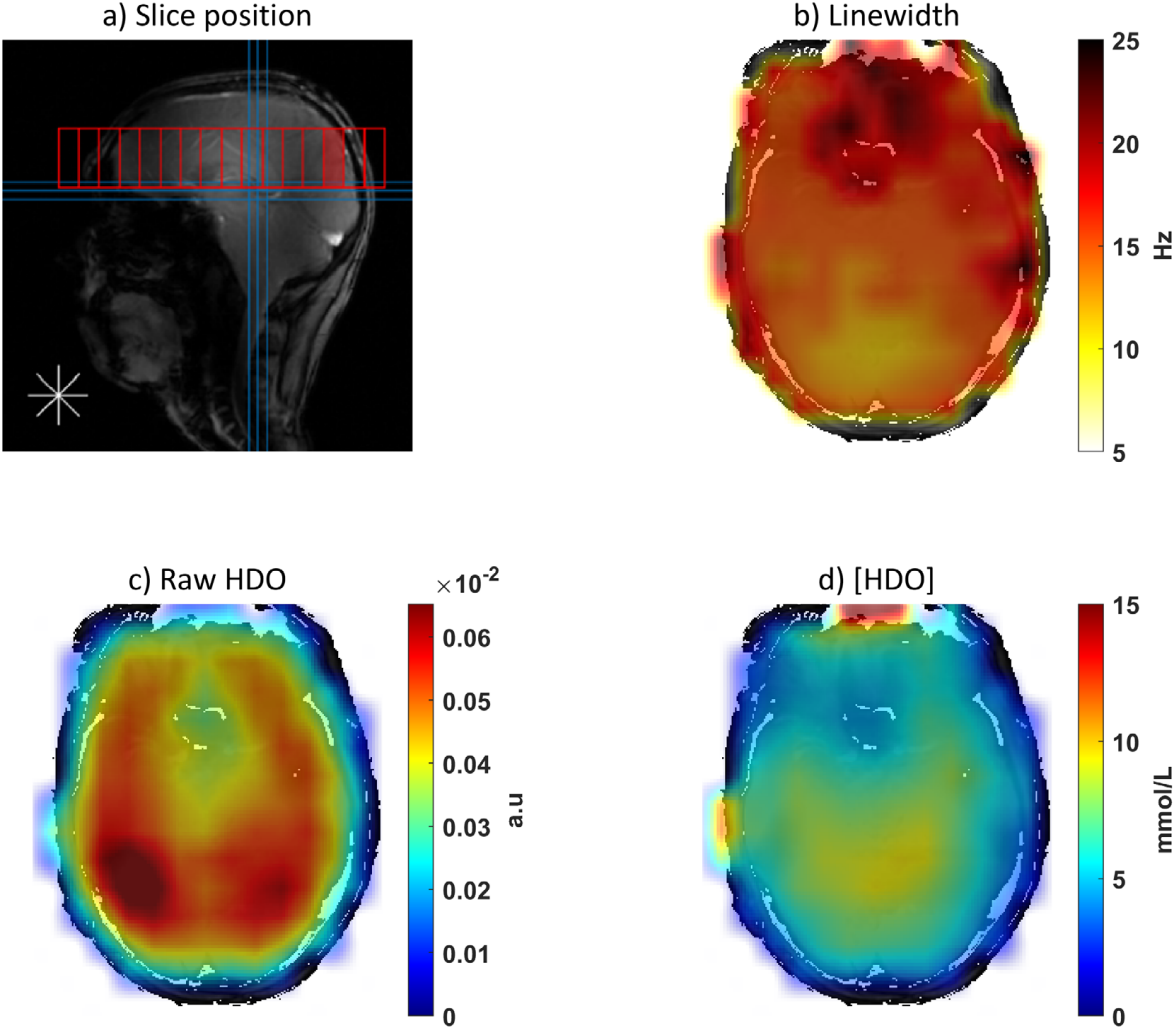
Natural HDO abundance scan in a healthy volunteer. (a) Position of the transverse slice. (b) Linewidth (FWHM) map, illustrating increased linewidth in the frontal lobe near the sinuses. (c) Raw HDO map (i.e., not calibrated, prior to absolute quantification). (d) Calibrated map showing absolute [HDO].

Sample HDO maps from natural abundance scans in one healthy volunteer are shown in Figure 4. Mean Linewidths across volunteers varied from 11 to 26 Hz in the brain. The strong signal peaks in posterior brain due to high receive sensitivity as seen in Figure 4c are corrected yielding a much more biologically plausible concentration distribution (Figure 4d).

**FIGURE 5:**
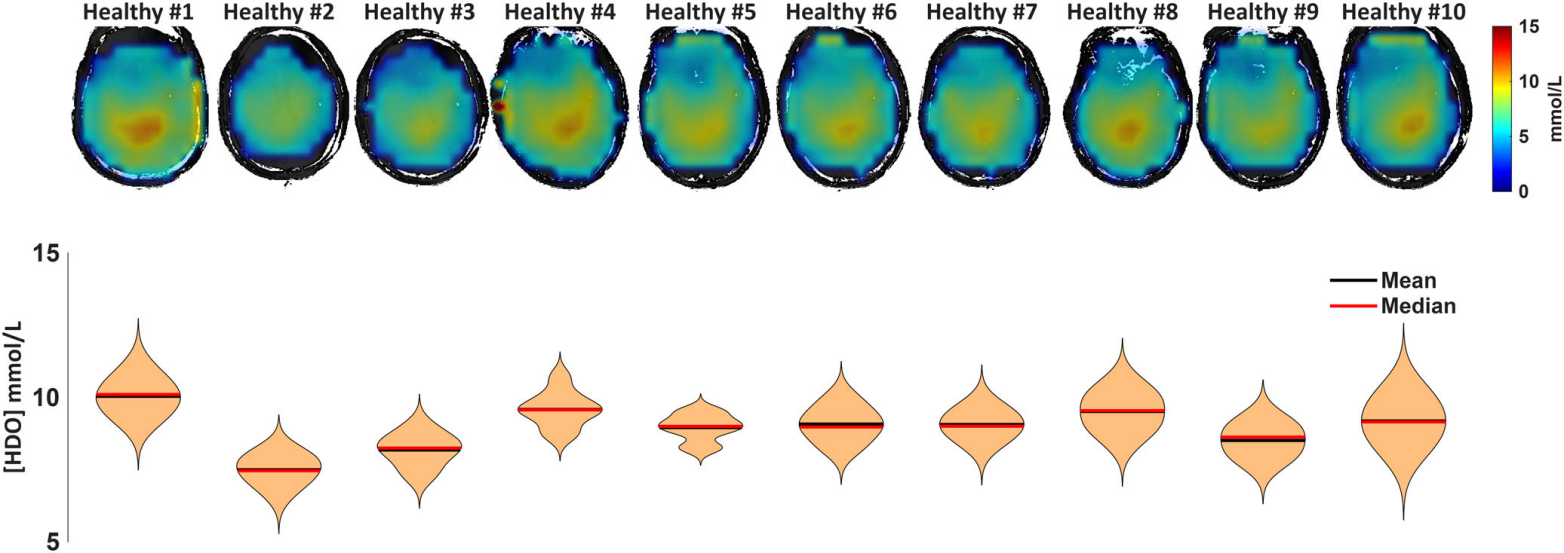
[HDO] maps for all healthy volunteers scanned with natural abundance (1st row). Distribution of the [HDO] values within the whole 3D brain (2nd row) shown as violin plots.

Figure 5 shows natural [HDO] maps for all volunteers, with violin plots depicting the distribution of [HDO] across the brain. The average HDO concentration in the brain in the 10 healthy volunteers was 8.96 ± 0.7 mmol/L (range 7.5–10.1 mmol/L).

#### 4.2.2 Imaging Glucose Metabolism in Healthy Controls

**FIGURE 6:**
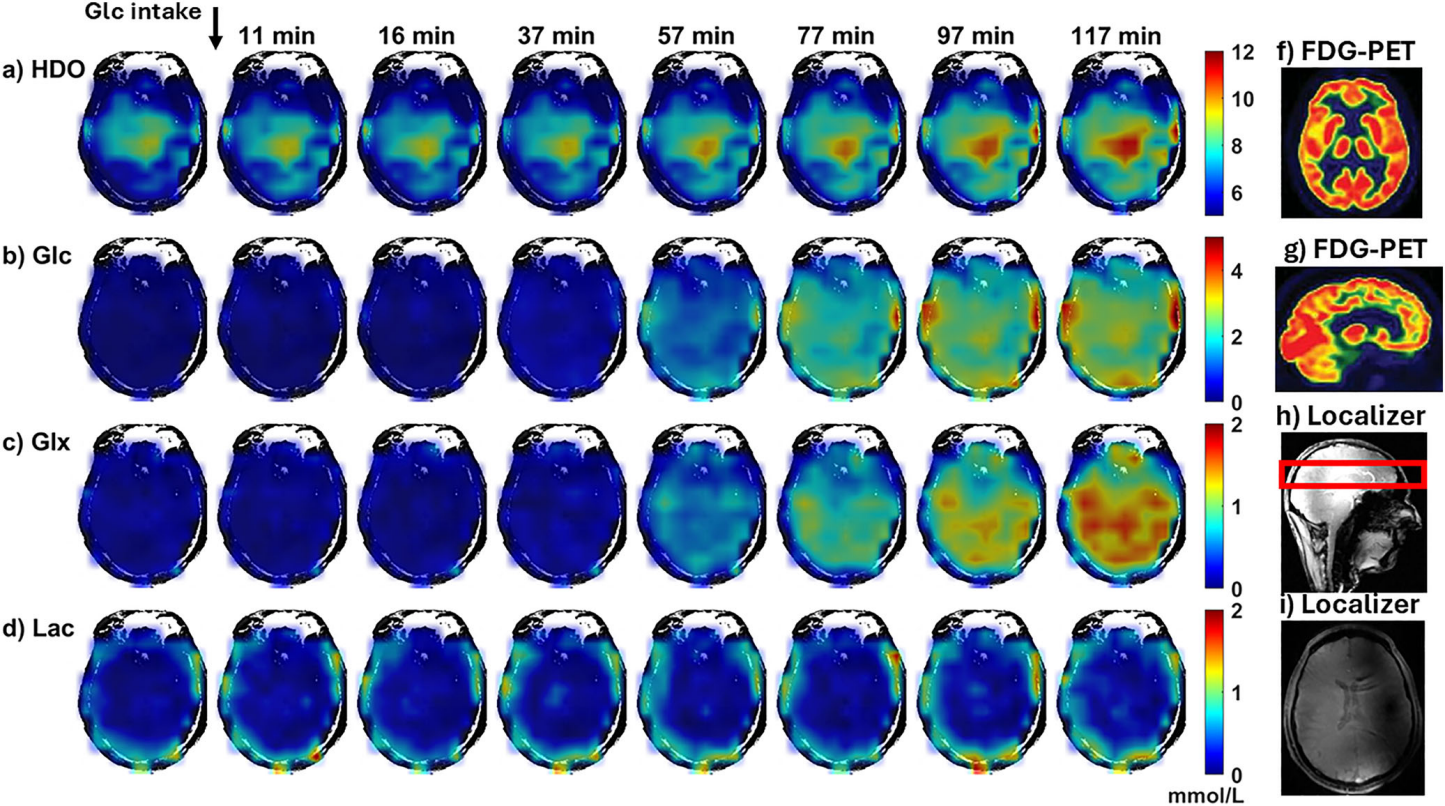
Evolution of metabolite maps evolution at baseline and following oral [6,6‐^2^H_2_]‐glucose administration to a healthy volunteer. HDO, Glc, Glx and Lac maps are shown in (a), (b), (c), and (d), respectively after application of the whole brain mask #1. Baseline DMI scans were acquired only in healthy volunteers. This was only done in healthy volunteers so that the patients didn't have to enter the magnet twice. Transaxial (f) and sagittal (g) ^18^F‐FDG PET images of a normal subject are shown, reproduced from Berti et al., PET Clinics, 2014 [72], Figure 2, with permission, alongside a sagittal (h) and transverse (i) GRE image for this volunteer.

^2^H images from a healthy volunteer after oral [6,6‐^2^H_2_]‐glucose are shown in Figure 6. Maps of HDO (Figure 6a), Glc (Figure 6b), Glx (Figure 6c), and Lac (Figure 6d) over time are presented. Signals from HDO, Glc, and Glx increased, with higher HDO in the brain center. For comparison, transverse (Figure 6f) and sagittal (Figure 6g) ^18^F‐FDG PET images of a normal subject are also shown, reproduced from Berti et al. [72]. As in prior DMI studies, a peripheral “Lac” ring is visible, which is said to be an artifact from subcutaneous lipids [53].

#### 4.2.3 Imaging Glucose Metabolism in Patients With Glioblastoma

**FIGURE 7:**
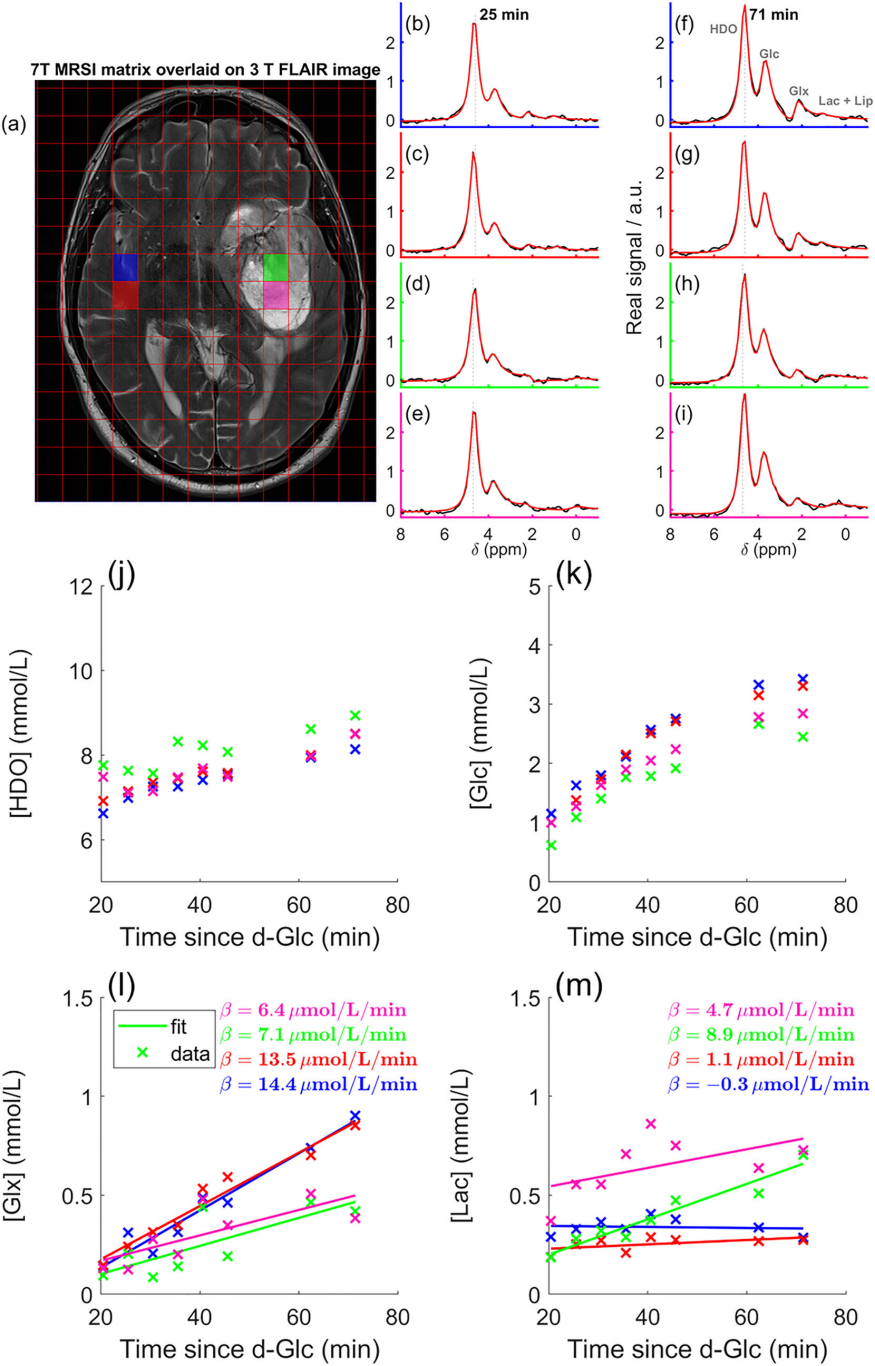
^2^H‐MRS data from a newly diagnosed glioblastoma patient. Spectra for 2 different time points from the highlighted voxels in control (green, light‐blue) and tumor (red, pink) regions. Spectra from 25 min (b–e) and 71 min (f–i) are shown from the highlighted voxels in (a), overlaid on an anatomical 3T image. For visualization (not fitting) spectra were apodized with a 20 Hz exponential filter. Time courses for each metabolite are shown in (i–m) following Glc administration. Linear fitted d[Glx]/dt and d[Lac]/dt from 20 to 90 min according to Equation (7) are shown in (l) and (m). Note that we trialed some more advanced sequences (not shown), so there is a gap in the time series (j–m).

**FIGURE 8:**
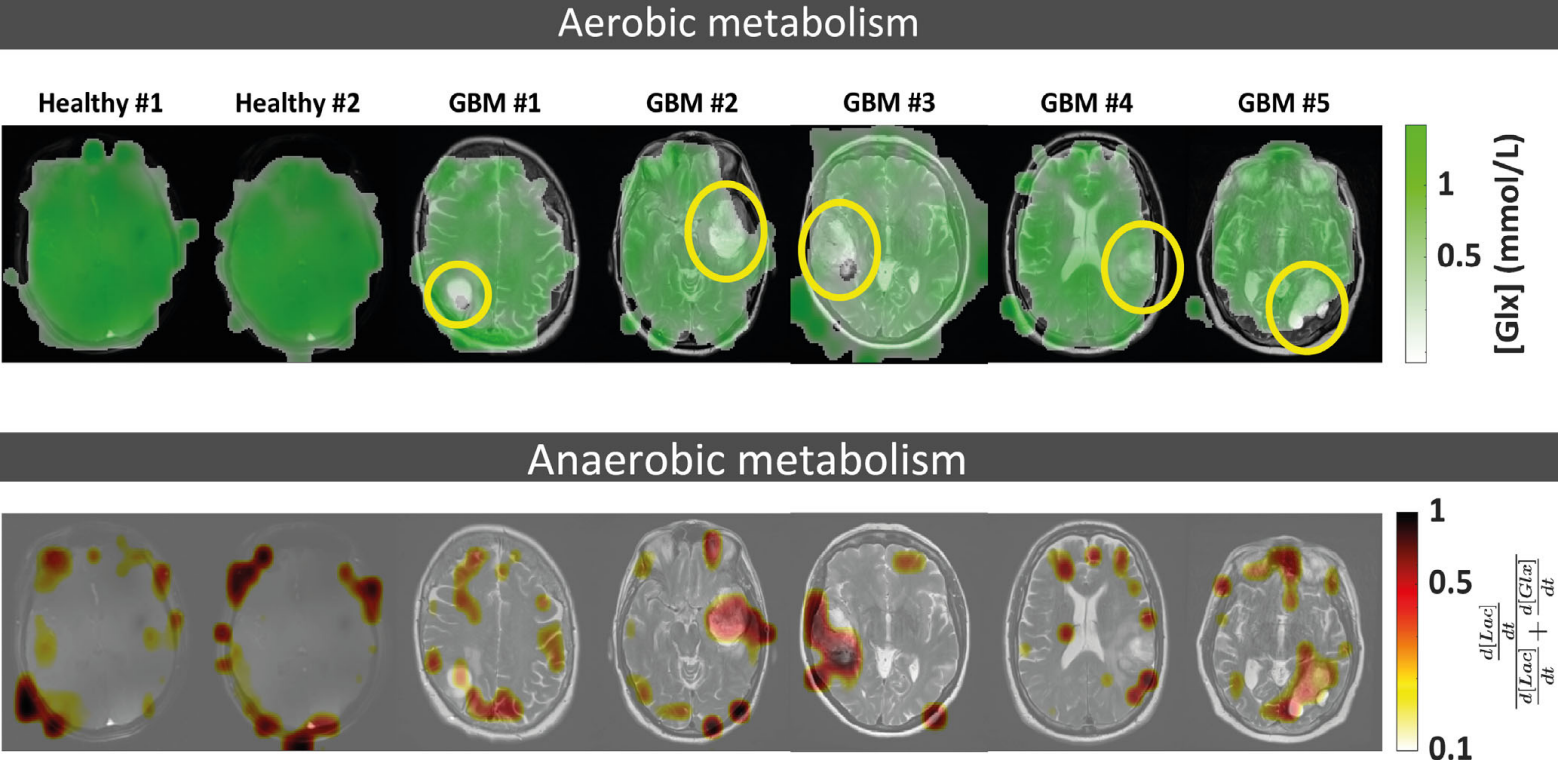
Maps of [Glx] (mmol/L) (top row, based on mask) and the $S_{\mathrm{Lac}}$ ratio (bottom row) for the two healthy volunteers and the 5 glioblastoma patients (based on mask #2). Tumor regions are circled in light yellow.

**FIGURE 9:**
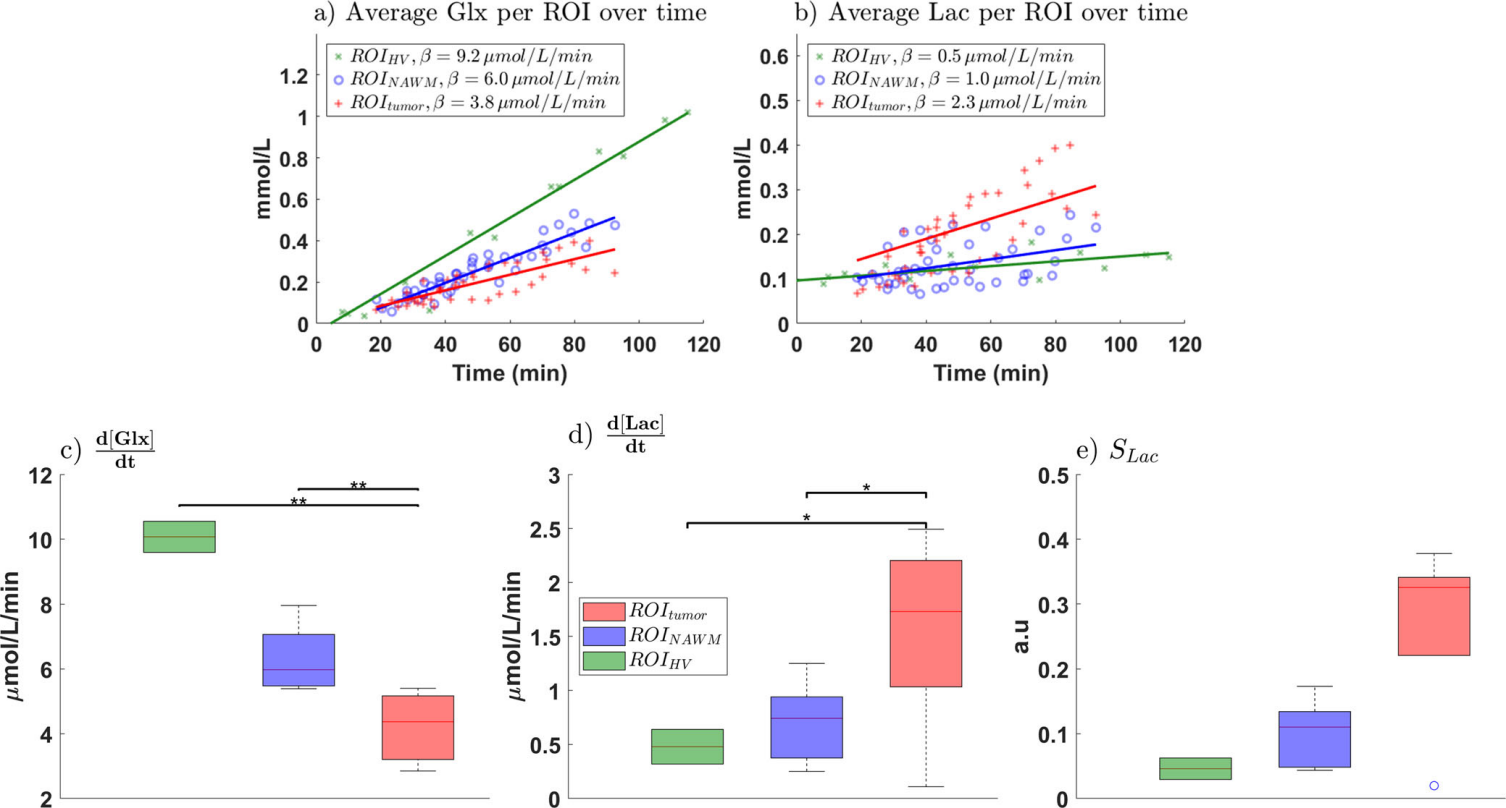
Comparison of Glx and Lac production in healthy volunteers and glioblastoma patients based the different ROis. (a) Glx and (b) Lac concentrations. Each data point represents an individual measurement per region (ROI), per time point (Section 3.7.2) from a volunteer or patient, and all data within each group/region were fitted with a linear model (healthy volunteers, ROI_HV_ = green; glioblastoma normal‐appearing white matter, ROI_NAWM_ = blue; tumor region, ROI_Tumor_ = red). Linear fits are also shown for each region. Rates of Glx (c), Lac (d) and $S_{\mathrm{Lac}}$ (e) production in each region (* = significant difference, *p* < 0.01, ** = highly significant, *p* < 0.001).

**FIGURE 10:**
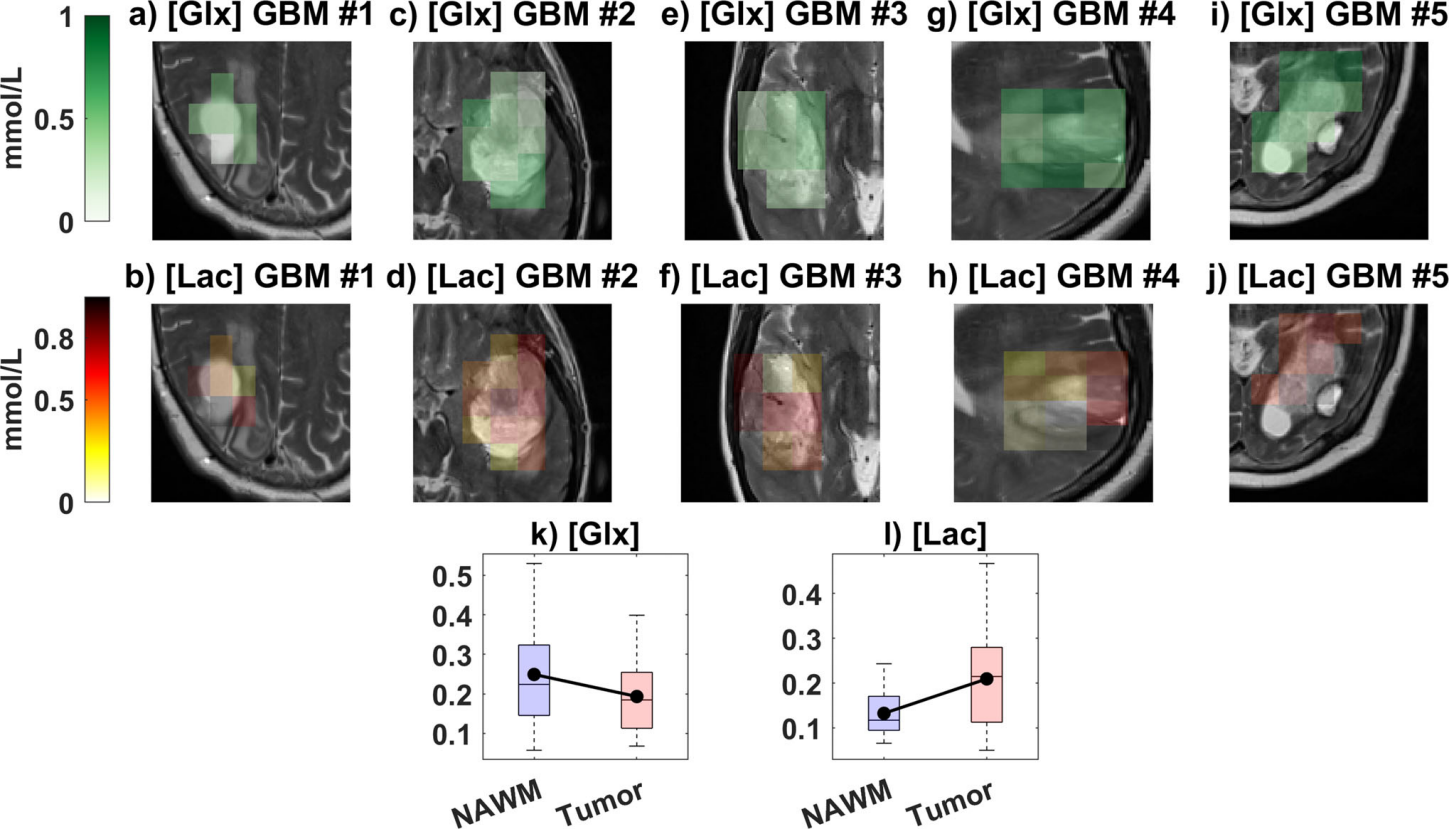
Metabolic heterogeneity in glioblastoma. [Glx] (a, c, e, g, i) and [Lac] (b, d, f, h, j) maps in all glioblastoma patients at the last time point. Boxplots showing concentrations of (k) Glx and (l) Lac for all 5 patients in NAWM and tumor regions. Black lines connect group means. For Glx, linear mixed modeling showed a significant effect of Region (*β* = −0.056, SE = 0.009, 95% CI [−0.073, −0.039], *p* < 0.001). For Lac, concentrations were significantly higher in tumor regions (*β* = 0.077, SE = 0.011, 95% CI [0.056, 0.098], *p* < 0.001). Each data point used for the boxplots corresponds to the average concentration within all voxels of the ROI (tumor or NAWM) for a given patient at a given time point, that is, one data point per region per patient per time point.

Images from glioblastoma patients are presented in Figures 7, 8, 9, 10. Example spectra from a tumor region and a control region are shown in Figure 7b–i. Deuterated metabolite time courses for the respective voxels are shown in Figure 7j–m. Increasing HDO, Glc and Glx concentrations were observed following the administration of labeled glucose. A higher Lac concentration uptake in the tumor can be observed in Figure 7m and an opposite trend can be seen for the Glx concentration (Figure 7l).

Figure 8 shows the Glx concentration maps (top row) derived from the last 3 time points for one healthy volunteer and the five glioblastoma patients. Also shown are maps of the relative metabolic flux toward lactate $S_{\mathrm{Lac}}$ ratio (bottom row). A lower Glx signal and a higher $S_{\mathrm{Lac}}$ ratio was observed in the tumor when compared to normal appearing brain tissue. Glc and Lac concentration maps are also shown in Figure SI3. Figure SI4 illustrates the benefits of applying an additional mask (mask #2), which excludes voxels with CRLB values of the ratio greater than 30%.

These findings are further supported by averaged voxel data from healthy volunteers (ROI_HV_), and glioblastoma patients in control (ROI_NAWM_) and tumor (ROI_Tumor_) regions (Figure 9a,b).

For Glx, the fitted rate of increase (d[Glx]/dt) differed significantly between tissue types. Tumor regions showed lower production rates (3.8 μmol/L/min, SE = 0.44; 95% CI: 2.88–4.66) compared with NAWM (6.0 μmol/L/min, SE = 0.36; 95% CI: 5.29–6.75; *p* < 0.001) and healthy volunteers (9.2 μmol/L/min, SE = 0.61; 95% CI: 7.86–10.53; *p* < 0.001).

For Lac, the fitted rate of increase (d[Lac]/dt) was significantly higher in tumors (2.3 μmol/L/min, SE = 0.87; 95% CI: 0.50–4.02) compared with NAWM (1.0 μmol/L/min, SE = 0.36; 95% CI: 0.28–1.74; *p* < 0.01) and healthy volunteers (0.5 μmol/L/min, SE = 0.17; 95% CI: 0.17–0.91; *p* < 0.01).

Overall, Glx production rates decreased from healthy tissue to NAWM and further to tumors (Figure 9c), whereas Lac production showed the inverse pattern (Figure 9d). The ratio $S_{\mathrm{Lac}}$ similarly increased in tumor tissue (Figure 9e).

#### 4.2.4 Metabolic Heterogeneity

Pairwise comparisons of tumor (ROI_Tumor_) and normal‐appearing regions (ROI_NAWM_) revealed significant differences in both Glx and Lac (Glx: *p* < 0.001, *z* = 4.6, *r* = 0.7; Lac: *p* < 0.001, *z* = −4.4, *r* = −0.67). Mean regional differences were −0.06 mmol/L for Glx and + 0.08 mmol/L for Lac.

The best models selected by a stepwise linear modeling approach were:

(10) [m]∼1+time+Region+PatientID

with Region emerging as a significant predictor for both metabolites. For Glx, Region was associated with lower concentrations in tumors (*p* < 0.001, *β* = −0.06 ± 0.01 mmol/L, 95% CI: −0.07 to −0.04; R^2^ = 0.876). For Lac, tumors showed higher concentrations (*p* < 0.001, *β* = 0.08 ± 0.01 mmol/L, 95% CI: 0.05–0.10; R^2^ = 0.759) (Figures SI5 and SI6, Figure 10k,l).

A linear mixed‐effects model (Model 1, Equation 10) further confirmed Region as a significant determinant, with random intercepts and slopes by patient (Glx: F (1, 84) = 49.5, *p* < 0.001, *β* = −0.06; Lac: F (1, 84) = 53.8, *p* < 0.001, *β* = +0.08).

Figure 10a–j illustrate spatial heterogeneity in three representative patients. Across all patients at the last time point, mean tumor concentrations were 0.5 ± 0.2 mmol/L for [Glx] (0.02–0.9 mmol/L range) and 0.5 ± 0.2 mmol/L for [Lac] (0.1–1.2 mmol/L range).

Likelihood ratio tests indicated that adding time as a random effect significantly improved model fit over random‐intercept‐only models (Model 2 vs. Model 3, Equation 8 vs. Equation 9). For Glx, random slopes yielded a better fit (*p* < 0.01, LR = 12.8), and for Lac, a similar improvement was observed (*p* < 0.01, LR = 4.6).

Accordingly, linear mixed‐effects models with random intercepts and slopes for time (Model 3, Equation 9) were fitted separately for Glx and Lac using tumor‐only data. These models included time as a fixed effect while capturing inter‐patient variability in both baseline levels and temporal trajectories.

For Glx, there was significant variability in intercepts (SD = 0.061, 95% CI: 0.026–0.145) and slopes (SD = 0.00098, 95% CI: 0.00046–0.00208). A strong negative intercept–slope correlation (*r* = −0.996) indicated that patients with higher baseline Glx tended to show slower—or even negative—temporal changes.

For Lac, variability was also evident in intercepts (SD = 0.077) and slopes (SD = 0.00069), though confidence intervals could not be reliably estimated due to limited patient numbers. Nevertheless, the model suggests meaningful differences in lactate dynamics across patients.

Together, these results confirm that there can be significantly different metabolic trajectories within tumor regions between individual glioblastoma patients, which is expected given the underlying biology of these tumors.

## 5 Discussion

### 5.1 Apparent Variation in Natural Abundance [HDO]

The absolute HDO concentration measured in the brain of 10 volunteers was lower than has been reported previously (10.12 to 17.8 mmol/L [30, 56, 73, 74]). However, to our knowledge, ours is the first study to report a directly calculated natural abundance HDO concentration in the human brain in vivo. Previous studies instead estimated brain HDO concentration based on the expected fraction of deuterated water in water (0.0115% [75] to 0.015% [76]) based on ^2^H natural abundance, combining this with assumed brain water fractions (ranging from 75% to 100%). Yet the fraction of deuterated water in water can vary depending on the environment [76, 77] and it is well established that the water content in brain tissue varies between approximately 70%–80% in gray and white matter [78]. Based on these values, the natural HDO concentration in the brain is estimated to range between 8.9 mmol/L (assuming 0.0115% deuterated water and 70% brain water fraction) and 13.3 mmol/L (assuming 0.015% deuterated water and 80% brain water fraction). The mean value measured in this study falls within this range.

Both the deuterium content of drinking water and tissue water fraction have been proposed as potential sources of inter‐subject variability. Deuterium abundance in tap water varies 15% across the USA and 5% within our county Cambridgeshire [79, 80]. Using reported ranges of gray‐ and white‐matter water content [81] and reported variability in tissue composition (GM%, WM%, CSF%), we estimate a 1%–2% contribution to inter‐individual differences in HDO concentration.

This suggests that a substantial fraction of observed variance in [HDO] in our healthy volunteer scans is likely due to technical factors affecting the measurement. Phantom‐replacement‐based absolute quantification is sensitive to coil loading, which varies between subjects depending on head size, anatomy, and positioning. Consistent with this interpretation, we observed a correlation between measured natural‐abundance HDO concentration and head size across volunteers. That suggests that much of the variability in [HDO] that we see may reflect the reproducibility limits of phantom‐based calibration for 7T DMI.

We recommend that future studies take blood at baseline and measure plasma [HDO] by mass spectrometry to check whether there are genuine inter‐subject differences in [HDO] at baseline.

Absolute quantification of metabolites provides direct concentration values, unlike metabolite ratios, which rely on reference metabolites that may themselves vary depending on the biochemical state of the tissue. This is particularly important in heterogeneous diseases like glioblastoma, where reference metabolites can be altered by tumor metabolism, edema, or treatment [14, 82] as we demonstrated by the changes in “normal‐appearing” tissue.

Moreover, absolute quantification avoids reliance on fixed assumptions, for example an assumption that the natural abundance HDO concentration is the same in all patients. Such assumptions fail to account for inter‐subject variability due to differences in hydration status, pathology, or metabolic state.

### 5.2 In Vivo Glucose Metabolism

A linear model was fitted to the Glx and Lac concentration time courses, consistent with approaches used in other studies [36, 56]. We chose this simple model because Glx and Lac concentrations did not reach a steady state; however, more sophisticated kinetic models are available for fitting concentration dynamics over time [83]. The elevated HDO and Glc signals observed in the center of the slice in Figure 6a might reflect high glucose uptake and subsequent metabolism in the basal ganglia, which is known from ^18^FDG‐PET imaging studies [72], plotted in Figure 6f,g.

Alternatively, it could reflect signal from CSF. Although CSF itself has negligible glucose metabolism in healthy people, HDO produced by glucose metabolism in other tissues would equilibrate from blood into CSF through well‐established water exchange pathways possibly delayed since the initial glucose bolus [84].

We used a UTE‐CSI sequence [85] optimized for DMI. However, newer sequences such as SSFP [12] or EPSI [37] may improve the signal‐to‐noise ratio and therefore the metabolic information attainable. The spatial resolution reported here falls between those reported in recent in vivo brain DMI studies at 7T (2–3 cm^3^) and 9.4T (1–2 cm^3^) [27, 28, 30, 35, 53, 55].

### 5.3 Assessment of Tumor Metabolism

In 4 of 5 glioblastoma patients, tumors showed an increased $S_{\mathrm{Lac}}$ratio when compared to normal appearing brain regions, indicating increased glycolytic metabolism, as has been observed in preclinical [83] and clinical studies [23]. Rates of Lac production varied between patients, consistent with preclinical studies showing the presence of different metabolic subtypes of glioblastoma with differences in glycolytic and oxidative activities [23, 83] and clinical studies showing that the tumors of glioblastoma patients can show differences in the proportion of these different metabolic subtypes [14]. Similar inter‐patient variation has also been observed in hyperpolarized [1‐^13^C]‐pyruvate studies, where tumor lactate labelling differed markedly [86].

The mean [Glx] increase rate in healthy volunteers is lower than the rate of 21 μmol/L/min reported in a recent study [36], but is comparable to other preclinical studies [14, 83].

Prior studies have suggested that [Glx] declines (5%–15% per decade) with aging [87, 88], which could partially contribute to the difference in concentrations seen between healthy volunteers (24–28 years old) and glioblastoma patients (> 50 years old).

Intra‐tumoral variations in [Glx] and [Lac] were also visible in all representative patients. Elevated lactate or high pyruvate to lactate conversion has been observed in hypoxic, poorly perfused regions (tumor rim vs. core) [86], while [Glx] changes are often found at invasive margins, with the direction of change depending on tumor subtype, local neuron–astrocyte metabolism and partial‐volume effects [89, 90, 91]. Moreover, intratumoral [Glx] heterogeneity appears to be associated with more aggressive tumor behavior, as seen in a recent study [91]. Heterogeneous glucose turnover has been observed previously where some tumor regions have higher glycolytic flux while others rely more on oxidative metabolism (OXPHOS) [83, 92]. For this reason, achieving higher spatial resolution in DMI would be valuable, and might be facilitated by advanced analysis methods such as deep learning, low‐rank approaches, or model‐based reconstruction techniques [73, 93].

Most glioblastoma studies have focused on [Lac] or [Lac]/[X] ratios. But these ratios are difficult to interpret when both numerator and denominator approach zero, often yielding misleading values. Our use of absolute quantification enables robust detection of [Glx] differences, highlighting [Glx] as a potentially more reliable biomarker of tumor heterogeneity in DMI. Future work should emphasize quantitative [Glx] analysis and consider full kinetic modeling to better capture fluxes, although this lies beyond the scope of the present study.

These metabolic heterogeneities in between tumors and patients could be due to the tumor microenvironment (TME), cellular composition, genetics or be driven by therapy effects [17, 19, 94].

The ability to assess these metabolic phenotypes non‐invasively in vivo with DMI could improve the precision of targeted therapies [14, 95].

### 5.4 Limitations

Our observations of intra‐ and inter‐tumor heterogeneity in glioblastomas are tempered by our small sample size. We encourage larger‐scale studies to confirm these findings with certainty.

We used a novel TEM head array coil for this brain 7T ^2^H‐MRSI study. B_1_^+^ maps acquired on a head‐shaped phantom showed acceptable coverage across the brain. The main limitation was B_1_^+^ inhomogeneity in frontal regions, particularly a modest B_1_^+^ drop‐out anterior‐right, although we note that this did not impede absolute quantification analyses.

Our choice of 16 receive elements was pragmatic: funding constraints required rapid procurement, so we adopted an existing mechanical design without optimizing the number or size of ^2^H receive elements. We do not claim that this configuration is superior to alternatives.

Absolute quantification relies on having similar phantom and in vivo B_1_ fields and noise covariance. We assumed this to be true, based on the chosen phantom recipe and experimental verification of its dielectric properties. Nevertheless, differences are possible especially for participants with small heads (Figures SI7–SI8). Future studies could consider direct measurement of B_1_^+^ in vivo using accelerated sequences [96, 97] and one could 3D print a smaller‐sized head to provide more representative calibration of B_1_^−^ fields for small‐headed participants.

Another limitation concerns the relatively broad range of lactate‐deuteron concentrations recovered in the multicompartment phantom experiment. We believe that this arises primarily from the limited spatial resolution of the CSI acquisition relative to the small lactate compartment. The 4‐cm diameter sphere spans only a few voxels in each dimension at the acquired resolution, leading to partial‐volume averaging (exacerbated by the voxel point‐spread‐function) between the lactate solution and the surrounding lower‐concentration HDO compartment. As a result, voxels intersecting the compartment boundary exhibit reduced apparent concentrations.

The slight overestimation in the center of the lactate phantom might reflect a local B_1_^+^ field enhancement due to increased conductivity in the lactate solution compared to the surrounding medium. Importantly, the mean concentration is close to the true value, indicating that despite voxel‐level variability, the overall quantification pipeline performs robustly when averaged over a region of interest. Higher‐resolution CSI or per‐scan rapid B_1_^+^ mapping might reduce these effects in future studies.

We did not sample the full glucose kinetics from the time of glucose administration through to steady state, as would typically be achieved with intravenous (IV) infusion protocols. Instead, our quantification relied on measurements acquired during the early post‐absorptive window, which we approximated with an effective first‐order initial reaction rate. This approach nevertheless captures the rising phase of metabolic labelling, during which incorporation into downstream metabolites (Glx and Lac) is most dynamic. We acknowledge that this limits full kinetic modeling, and that it could be confounded by the rate of glucose absorption from the gut. We recommend that future studies consider IV glucose administration, with regular blood sampling for mass spectrometric confirmation of D/H ratios and circulating deuterated metabolite concentrations.

While using T_1_ mapping to estimate the relative contributions of different tissue types in each voxel would be valuable, this was not possible in our study because only a GRE structural image was acquired, rather than a dedicated T_1_‐weighted image such as MP2RAGE. We could not reliably segment gray matter, white matter and CSF. Future studies may wish to incorporate a T_1_ mapping sequence.

Future studies using natural‐abundance HDO as a reference may wish to measure blood plasma [HDO] via mass spectrometry [98], because tap water [HDO] varies by up to 15% across the USA and by 5% across Cambridgeshire [80, 81, 99].

## 6 Conclusion

We present a method for absolute quantification of ^2^H MRS images, which we validated using a TEM ^2^H/^1^H head array coil on phantoms and healthy volunteers. Studies in glioblastoma patients revealed both inter‐ and intra‐tumor metabolic heterogeneity. We anticipate that this new method will enhance our understanding of brain metabolism and tumor behavior, as well as aid in optimizing drug therapies and monitoring treatment response.

## Funding

This work was supported by the EU Horizon Europe project “MITI” #101058229, NIHR Cambridge Biomedical Research Centre (NIHR203312), the NIHR Applied Research Collaboration East of England, Medical Research Council [UKRI2679; UKRI790], (MR N013433‐1), the Cambridge Trust, Commonwealth Scholarship Commission, Armstrong Fund and the European Union's H2020 research and innovation program under grant agreement [801075], CRUK Rosetta Grand Challenge grant (A25045).

## Conflicts of Interest

The authors declare no conflicts of interest.

## Supporting information


**Figure SI1:** Effects of the lights on the environment noise captured with the coil's receive channels. Difference in noise level between lights ON, lights OFF and circuit breaker turned OFF completely in the 7T “technical room”.
**Figure SI2:** Illustration of the different ROIs that have been chosen for further statistical analysis on (a) a glioblastoma patient (ROITumor and ROINAWM) and (b) a healthy volunteer (ROIHV).
**Figure SI3:** Maps of [Glc] (mmol/L) (top row) and [Lac] (bottom row) for the two healthy volunteers and the 5 glioblastoma patients based on mask #1 averaged across the last three time points.
**Figure SI4:** Maps of $S_{\mathrm{Lac}}$ratio (top row) for two healthy volunteers and five glioblastoma patients, based on mask #1 (i.e., without excluding voxels with CRLB values of the ratio > 30%). The bottom row shows the corresponding CRLB maps for the same subjects. Note that the % CRLB is highest in areas with low metabolic rate—i.e. low d[Lac]/dt and low d[Glx]/dt.
**Figure SI5:** Matlab code and output from the stepwise linear modeling of [Glx] using the Akaike Information Criterion (AIC).
**Figure SI6:** Matlab code and output from the stepwise linear modeling of [Lac] using the Akaike Information Criterion (AIC).
**Figure SI7:** B_1_^+^ maps measured using multiple CSI scans at different voltages (see Methods in paper for detail) for (A) the head‐shaped phantom as described in the Methods and (B) a 5.4 L carboy cylinder (17 cm diameter, shown in (C)) filled with 63 g of deuterium oxide, 23 g of sodium chloride, 1.25 mL of Dotarem and 10 g of sodium benzoate, with the rest of water.
**Figure SI8:** Variation of measured natural‐abundance [HDO] as a function of brain volume for all 10 volunteers (slope = 0.0047 mmol/L/cm^3^, 95% CI = [−0.0005, 0.0098]). An additional fit was also performed by removing a potential outlier (smallest brain size) with a slope of 0.0017 mmol/L/cm^3^ and 95% CI = [−0.0050, 0.0084]. Brain volumes were extracted using the Brain Extraction Tool (BET) implemented in the FMRIB Software Library (FSL). The shaded gray and blue areas represent the difference in deuterium enrichment variability on a continent (US) scale compared to a regional scale.

## Data Availability

The code is hosted on GitLab and can be made available upon request to the authors. Data is available from the corresponding author subject to a data transfer agreement as mandated by our ethical approvals and hospital policies.
