# Supplementary material for: Absolute Quantification of Brain Deuterium Metabolic Imaging in Healthy Volunteers and Glioblastoma Patients at 7T
Source: Magn Reson Med. 2026 Feb 23;95(6):3077–94. doi: 10.1002/mrm.70308 (PMC13049263; doi:10.1002/mrm.70308)
Supplement: Supplementary file 1 — Figure SI1: Effects of the lights on the environment noise captured with the coil's receive channels. Difference in noise level between lights ON, lights OFF and circuit breaker turned OFF completely in the 7T “technical room”. Figure SI2: Illustration of the different ROIs that have been chosen for further statistical analysis on (a) a glioblastoma patient (ROITumor and ROINAWM) and (b) a healthy volunteer (ROIHV). Figure SI3: Maps of [Glc] (mmol/L) (top row) and [Lac] (bottom row) for the two healthy volunteers and the 5 glioblastoma patients based on mask #1 averaged across the last three time points. Figure SI4: Maps of SLacratio (top row) for two healthy volunteers and five glioblastoma patients, based on mask #1 (i.e., without excluding voxels with CRLB values of the ratio > 30%). The bottom row shows the corresponding CRLB maps for the same subjects. Note that the % CRLB is highest in areas with low metabolic rate—i.e. low d[Lac]/dt and low d[Glx]/dt. Figure SI5: Matlab code and output from the stepwise linear modeling of [Glx] using the Akaike Information Criterion (AIC). Figure SI6: Matlab code and output from the stepwise linear modeling of [Lac] using the Akaike Information Criterion (AIC). Figure SI7: B1 + maps measured using multiple CSI scans at different voltages (see Methods in paper for detail) for (A) the head‐shaped phantom as described in the Methods and (B) a 5.4 L carboy cylinder (17 cm diameter, shown in (C)) filled with 63 g of deuterium oxide, 23 g of sodium chloride, 1.25 mL of Dotarem and 10 g of sodium benzoate, with the rest of water. Figure SI8: Variation of measured natural‐abundance [HDO] as a function of brain volume for all 10 volunteers (slope = 0.0047 mmol/L/cm3, 95% CI = [−0.0005, 0.0098]). An additional fit was also performed by removing a potential outlier (smallest brain size) with a slope of 0.0017 mmol/L/cm3 and 95% CI = [−0.0050, 0.0084]. Brain volumes were extracted using the Brain Extraction Tool (BET) implemented in the [file MRM-95-3077-s001.docx]

# Supplementary information

## Performance of the coil array

We demonstrated the feasibility of using a novel TEM head array coil for brain ²H-MRSI at 7T. B₁⁺ maps acquired on a head-shaped phantom showed acceptable coverage across the brain. The main limitation is B₁⁺ inhomogeneity in frontal regions, particularly left–right asymmetry. The modest anterior-right B1+ drop-out did not impede absolute quantification analyses.

Transmit efficiency is comparable to values from previous 7T DMI studies (around 0.9 μT/√W)(1). Higher transmit efficiencies have been reported in other DMI setups, such as around 1.5/2 μT/√W at 7T(2,3) and 4 μT/√W at 9.4T(4).

A key advantage of this design is its tolerance of a “missing rung,” yielding an open geometry that improves patient comfort and will enable future visual-stimulus fMRS studies.

This coil enables interleaved ¹H/²H acquisitions(5-7). This provides the opportunity to implement motion correction, frequency shot-to-shot correction, and interleaved B₀ shimming(6,7). The main limitation is B₁⁺ inhomogeneity in frontal regions, particularly left–right asymmetry.

The ²H/¹H coil configuration used in this study was not the outcome of an optimisation study, but rather followed a mechanical design that could be manufactured rapidly to meet project timelines. Recent work has suggested that increasing the number of ²H receive channels does not always translate into proportional improvements in SNR or coverage(2). Therefore, the performance of alternative element counts and geometries remains an open question.


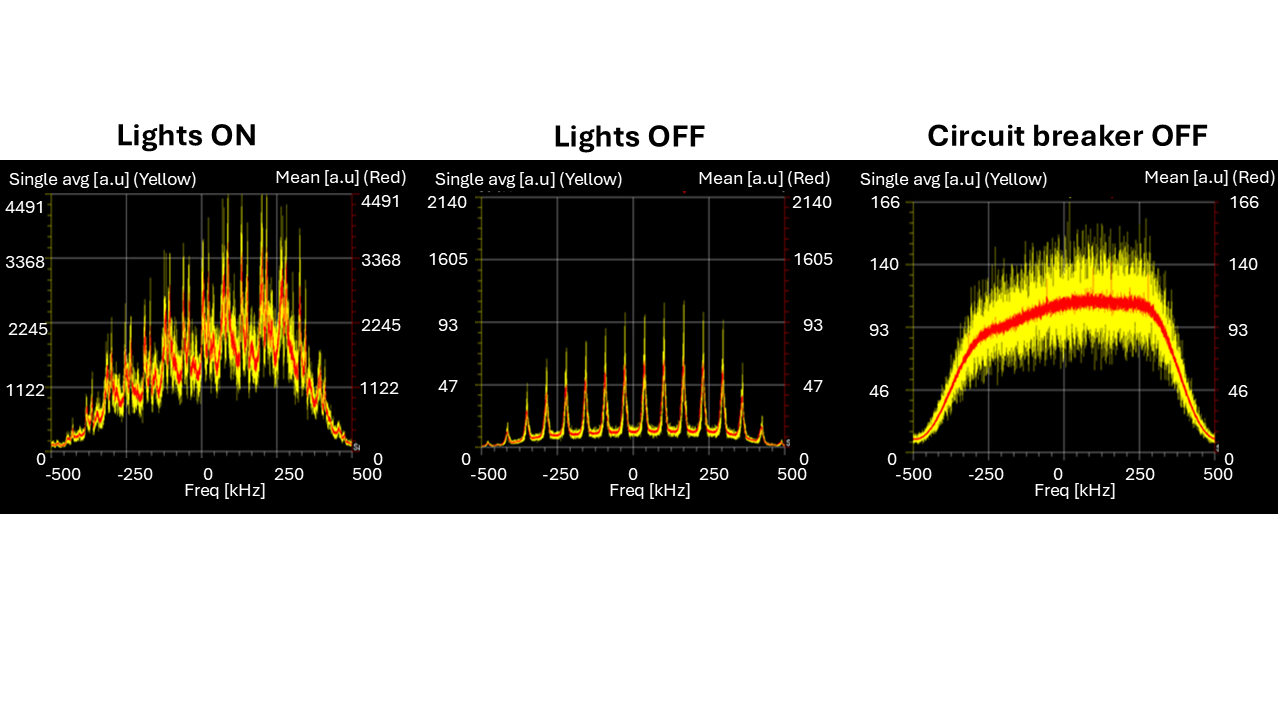


Figure SI1: Effects of the lights on the environment noise captured with the coil’s receive channels. Difference in noise level between lights ON, lights OFF and circuit breaker turned OFF completely in the 7T “technical room”.


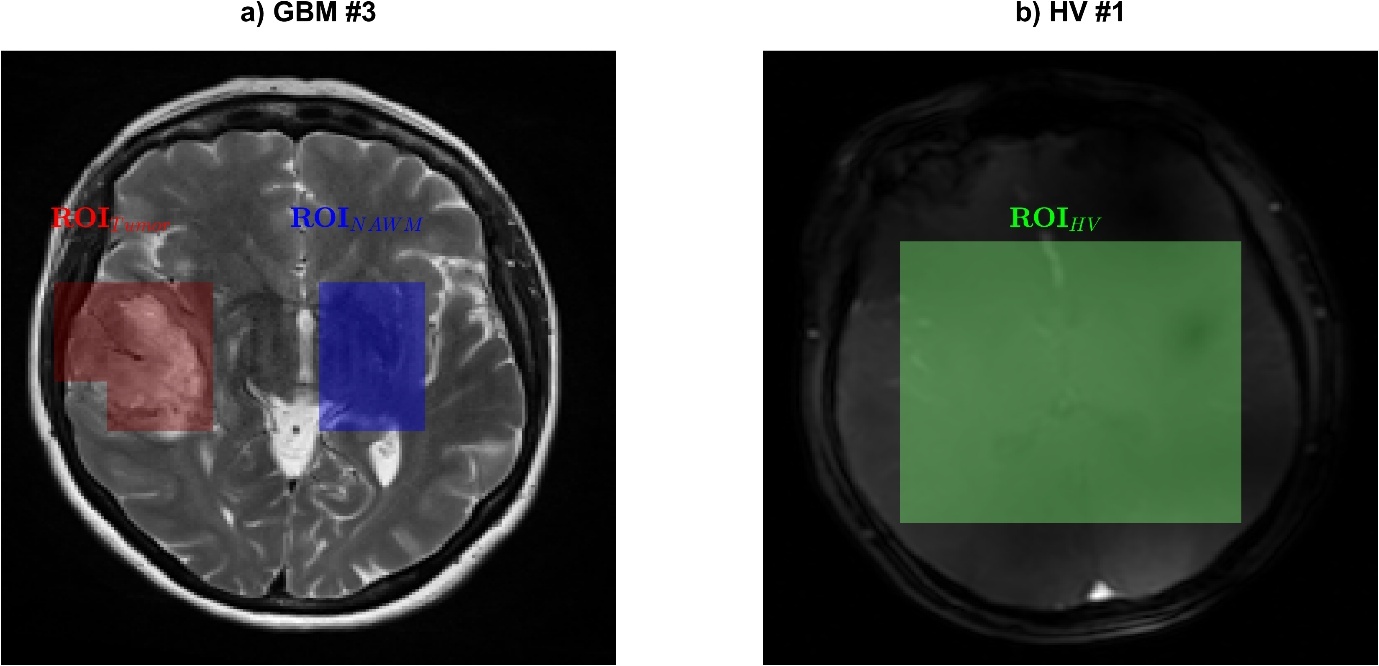


Figure SI2: Illustration of the different ROIs that have been chosen for further statistical analysis on a) a glioblastoma patient (ROI_Tumor_ and ROI_NAWM_) and b) a healthy volunteer (ROI_HV_).


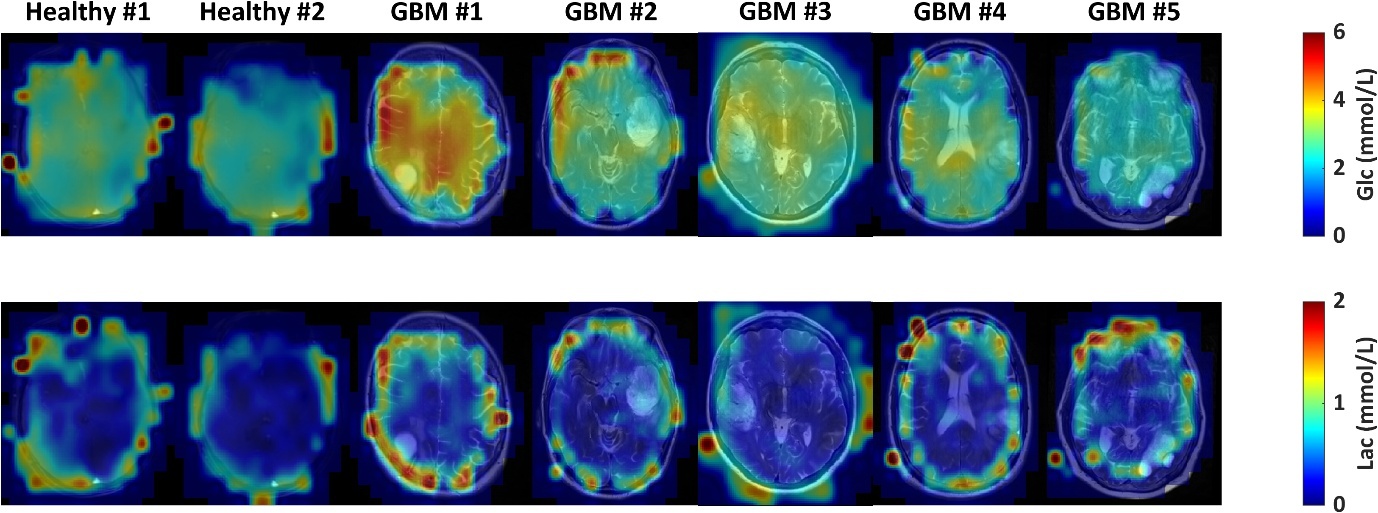


Figure SI3: Maps of [Glc] (mmol/L) (top row) and [Lac] (bottom row) for the two healthy volunteers and the 5 glioblastoma patients based on mask #1 averaged across the last three time points.


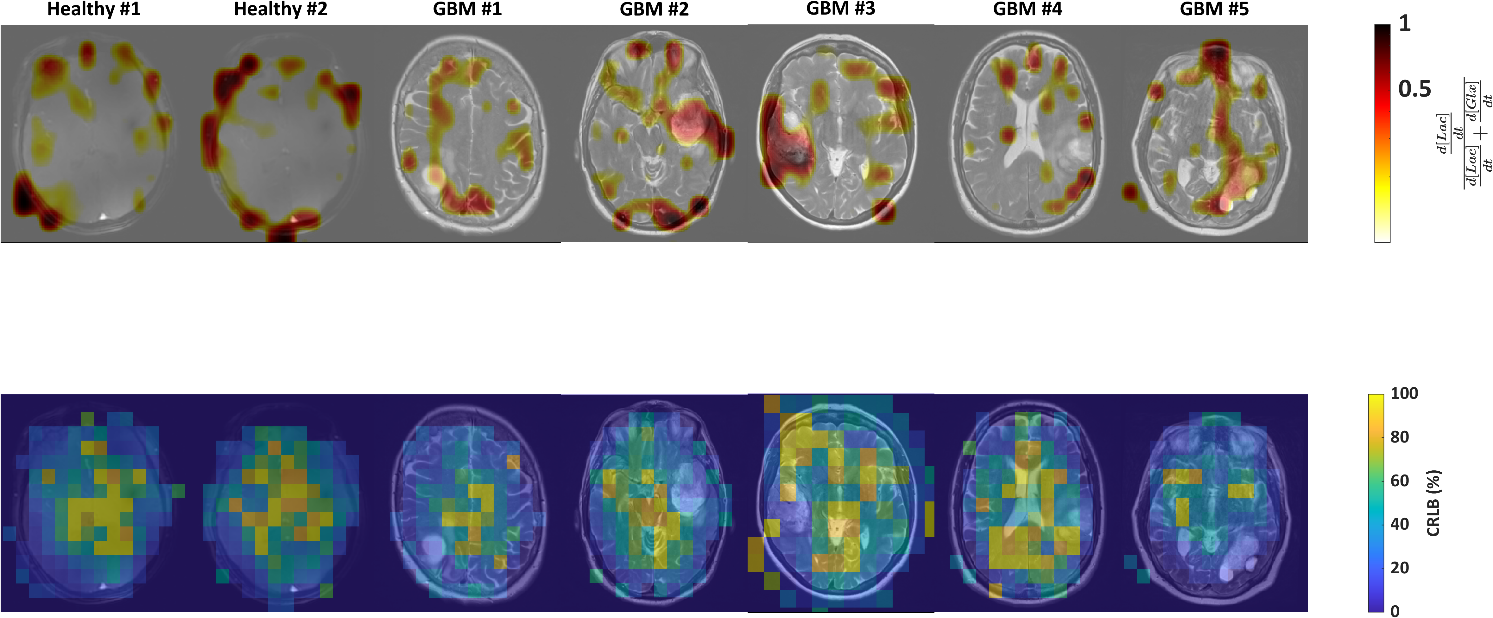


Figure SI4: Maps of $S_{\text{Lac}}$ratio (top row) for two healthy volunteers and five glioblastoma patients, based on mask #1 (i.e., without excluding voxels with CRLB values of the ratio >30%). The bottom row shows the corresponding CRLB maps for the same subjects. Note that the % CRLB is highest in areas with low metabolic rate -- i.e. low d[Lac]/dt and low d[Glx]/dt.


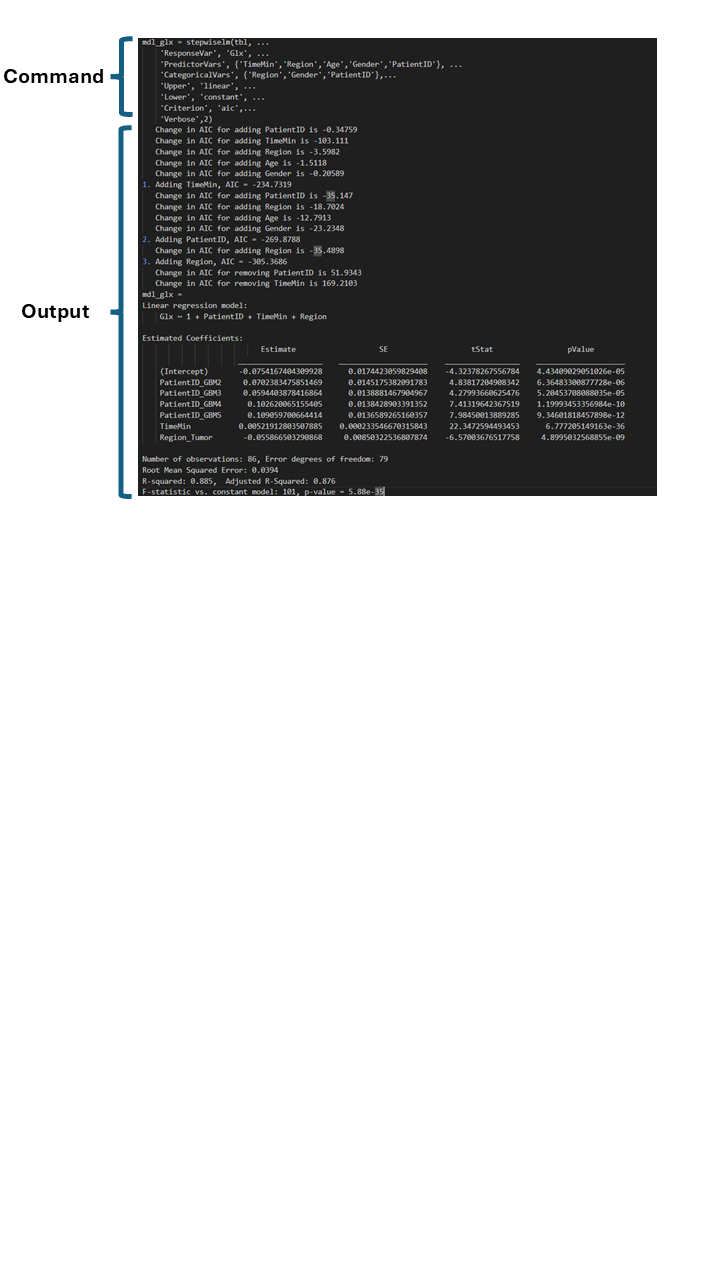


Figure SI5: Matlab code and output from the stepwise linear modelling of [Glx] using the Akaike Information Criterion (AIC).


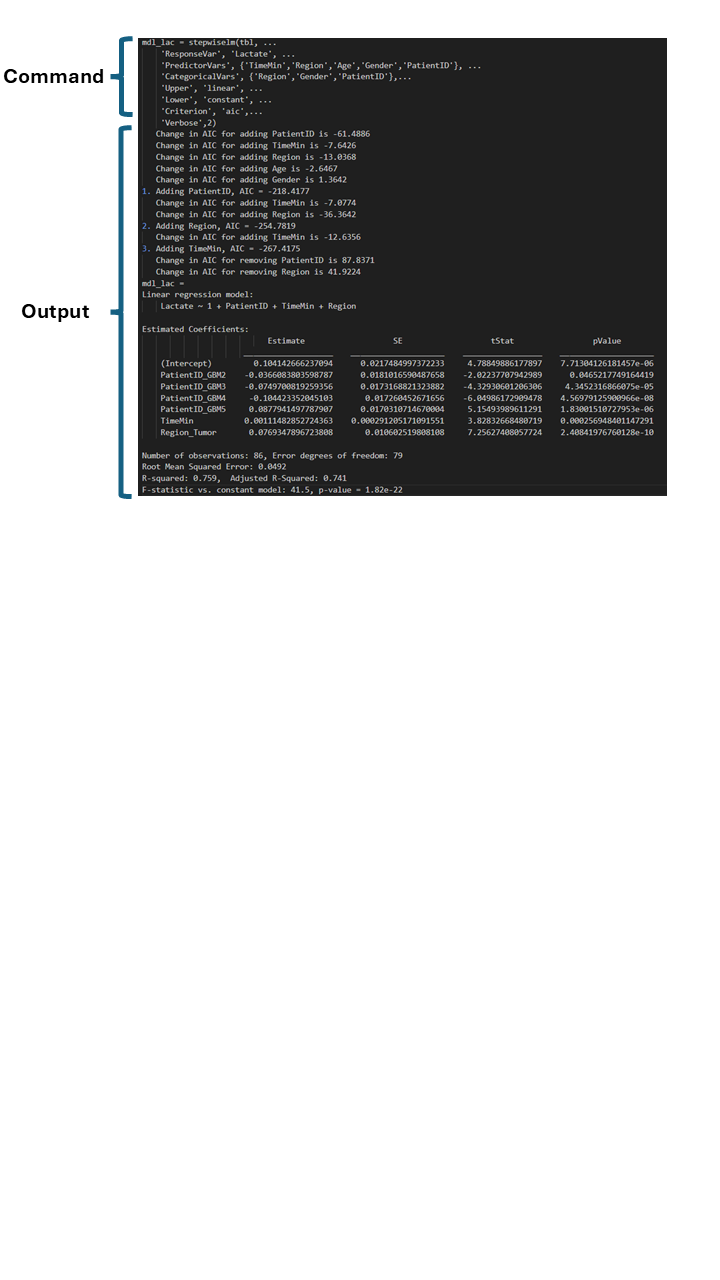


Figure SI6: Matlab code and output from the stepwise linear modelling of [Lac] using the Akaike Information Criterion (AIC).


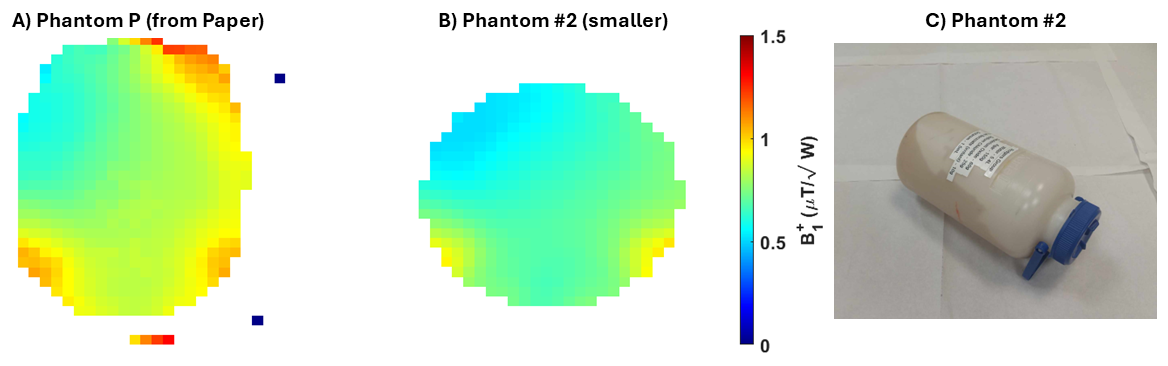


*Figure SI7: B_1_^+^ maps measured using multiple CSI scans at different voltages (see Methods in paper for detail) for A) the head-shaped phantom as described in the Methods and B) a 5.4 L carboy cylinder (17cm diameter, shown in C)) filled with 63 g of deuterium oxide, 23 g of sodium chloride, 1.25 mL of Dotarem and 10 g of sodium benzoate, with the rest of water.*


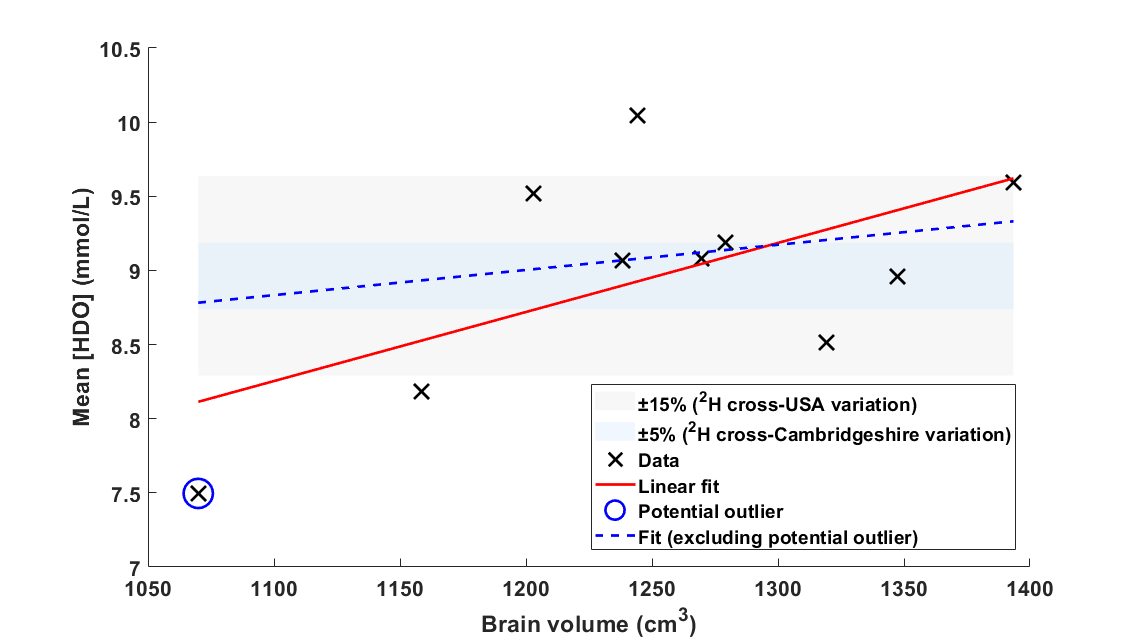


*Figure SI8: Variation of measured natural-abundance [HDO] as a function of brain volume for all 10 volunteers (slope = 0.0047 mmol/L/cm^3^, 95% CI = [- 0.0005, 0.0098]). An additional fit was also performed by removing a potential outlier (smallest brain size) with a slope of 0.0017 mmol/L/cm^3^ and 95% CI = [-0.0050, 0.0084]. Brain volumes were extracted using the Brain Extraction Tool (BET) implemented in the FMRIB Software Library (FSL). The shaded grey(8) and blue(9) areas represent the difference in deuterium enrichment variability on a continent (US) scale compared to a regional scale.*

# Additional References For Supplementary Information

1. Gursan A, Kahraman-Agir B, Gosselink M, Welting D, Froeling M, Hoogduin H, Wiegers EC, Prompers JJ, Klomp DWJ. Development of a Double Tuned (2)H/(31)P Whole-Body Birdcage Transmit Coil for (2)H and (31)P MR Applications From Head to Toe at 7 T. NMR Biomed 2025;38(3):e5325.

2. Li X, Zhu XH, Li Y, Wang T, Zhang G, Wiesner HM, Liang ZP, Chen W. Quantitative mapping of key glucose metabolic rates in the human brain using dynamic deuterium magnetic resonance spectroscopic imaging. PNAS Nexus 2025;4(3):pgaf072.

3. Seres Roig E, De Feyter HM, Nixon TW, Ruhm L, Nikulin AV, Scheffler K, Avdievich NI, Henning A, de Graaf RA. Deuterium metabolic imaging of the human brain in vivo at 7 T. Magn Reson Med 2023;89(1):29-39.

4. Zhang Y, Gao Y, Fang K, Ye J, Ruan Y, Yang X, Zhang Y, Thompson G, Chen G, Zhang X. Proton/Deuterium Magnetic Resonance Imaging of Rodents at 9.4T Using Birdcage Coils. Bioelectromagnetics 2022;43(1):40-46.

5. Karkouri J, Rodgers CT. Sequence building block for magnetic resonance spectroscopy on Siemens VE-series scanners. NMR Biomed 2024;37(10):e5165.

6. Liu Y, De Feyter HM, Fulbright RK, McIntyre S, Nixon TW, de Graaf RA. Interleaved fluid-attenuated inversion recovery (FLAIR) MRI and deuterium metabolic imaging (DMI) on human brain in vivo. Magn Reson Med 2022;88(1):28-37.

7. Liu Y, De Feyter HM, Corbin ZA, Fulbright RK, McIntyre S, Nixon TW, de Graaf RA. Parallel Detection of Multicontrast MRI and Deuterium Metabolic Imaging for Time-efficient Characterization of Neurologic Diseases. Radiology 2025;315(1):e241597.

8. Strekalova T, Evans M, Chernopiatko A, Couch Y, Costa-Nunes J, Cespuglio R, Chesson L, Vignisse J, Steinbusch HW, Anthony DC, Pomytkin I, Lesch KP. Deuterium content of water increases depression susceptibility: the potential role of a serotonin-related mechanism. Behav Brain Res 2015;277:237-244.

9. Rozanski K. Deuterium and oxygen-18 in European groundwaters — Links to atmospheric circulation in the past. Chemical Geology: Isotope Geoscience section 1985;52(3-4):349-363.
